# Recent Strides in the Transition Metal-Free Cross-Coupling of Haloacetylenes with Electron-Rich Heterocycles in Solid Media

**DOI:** 10.3390/molecules25112490

**Published:** 2020-05-27

**Authors:** Lyubov’ N. Sobenina, Boris A. Trofimov

**Affiliations:** A.E. Favorsky Irkutsk Institute of Chemistry, Siberian Branch, Russian Academy of Sciences, 1 Favorsky Str., 664033 Irkutsk, Russia; sobenina@irioch.irk.ru

**Keywords:** electrophilic haloacetylenes, pyrroles, ethynylpyrroles, furans, thiophenes, pyrazoles, Al_2_O_3_

## Abstract

The publications covering new, transition metal-free cross-coupling reactions of pyrroles with electrophilic haloacetylenes in solid medium of metal oxides and salts to regioselectively afford 2-ethynylpyrroles are discussed. The reactions proceed at room temperature without catalyst and base under solvent-free conditions. These ethynylation reactions seem to be particularly important, since the common Sonogashira coupling does not allow ethynylpyrroles with strong electron-withdrawing substituents at the acetylenic fragments to be synthesized. The results on the behavior of furans, thiophenes, and pyrazoles under the conditions of these reactions are also provided. The reactivity and structural peculiarities of nucleophilic addition to the activated acetylene moiety of the novel C-ethynylpyrroles are considered.

## 1. Introduction

Functionalized five-membered aromatic heterocycles represent a frequent structural motif of bioactive natural products and pharmaceuticals [1,2,3,4,5,6,7,8,9,10,11,12]. Among them, of particular interest are the compounds bearing acetylenic moieties [13,14,15]. The combination of the electron-rich, five-membered aromatic heterocyclic nucleus with highly reactive carbon-carbon triple bond in one molecule allows using these compounds for the targeted synthesis of various complex heterocyclic systems. Commonly, ethynylation of heterocycles is implemented via Sonogashira reaction employing the halogenated heterocycles and terminal alkynes [16,17,18,19]. In 2004, as a complementation to the existing cross-coupling protocols, the direct palladium- and copper-free ethynylation of the pyrrole ring with haloacetylenes in the Al_2_O_3_ medium (room temperature, no solvent) was discovered [20]. Later, haloacetylenes were involved in ethynylation of diverse heterocycles using palladium [21,22], nickel [23,24], copper [25], or gold [26] catalysts. In parallel, the Al_2_O_3_-mediated ethynylation of pyrroles and indoles kept being steadily developed [27,28,29,30,31,32,33,34,35,36,37,38,39,40,41,42,43]. A number of pyrroles with alkyl, cycloalkyl, aryl, and hetaryl substituents were successfully ethynylated with acylhaloacetylenes and halopropynoates within a framework of this cross-coupling procedure. It was shown that some other metal oxides (MgO, CaO, BaO) [33] and salts (K_2_CO_3_) [35] can also be beneficially applied instead of Al_2_O_3_. On the basis of the experimental facts, it was concluded that this cross-coupling includes the nucleophilic attack of pyrroles at the electron-deficient triple bond of haloacetylenes followed by the elimination of hydrogen halide from the zwitterionic intermediates.

As far as the relationship between the reactivity of the heterocycle and the solid salt used is concerned, a broad screening of various metal oxides and salts as mediators for the cross-coupling has shown that some of them are rather active (i.e., BaO). However, due to availability and convenience of the work-up of the reaction mixtures, Al_2_O_3_ and K_2_CO_3_ were taken as the agents of choice. A selection of specific metal oxides (Al_2_O_3_ or K_2_CO_3_) for a particular reaction is determined experimentally because the results significantly depend on the structure of both the pyrrole and haloacetylene employed.

This methodology was already partially documented in recent reviews [44,45,46,47,48,49,50,51,52]. This survey covers the recent publications (since 2014) concerning this reaction and the related chemistry which have not been yet summarized in a review.

## 2. Cross-Coupling of Haloacetylenes with Electron-Rich Heterocycles

### 2.1. Cross-Coupling of Haloacetylenes with Pyrroles

#### 2.1.1. Cross-Coupling of Bromo- and Iodopropiolaldehydes with Pyrroles

**Scheme 1:**
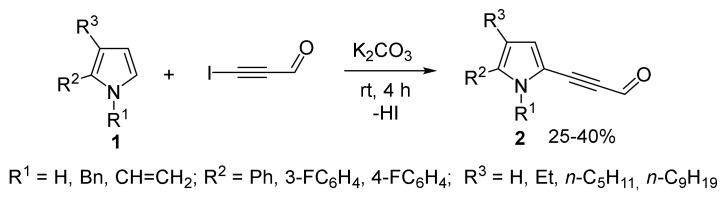
Synthesis of 3-(pyrrol-2-yl)propiolaldehydes **2**.

A series of substituted pyrroles **1** were ethynylated by iodopropiolaldehyde in solid K_2_CO_3_ (a 10-fold excess) under mild conditions without solvent to afford highly reactive functionalized pyrrole compounds, 3-(pyrrol-2-yl)propiolaldehydes **2**, in up to 40% yield (Scheme 1) [53]. The reagents were ground intensively for 5–10 min and allowed to stand at room temperature for 4 h. Iodopropioaldehyde was more preferable over explosive bromopropiolaldehyde.

**Scheme 2:**
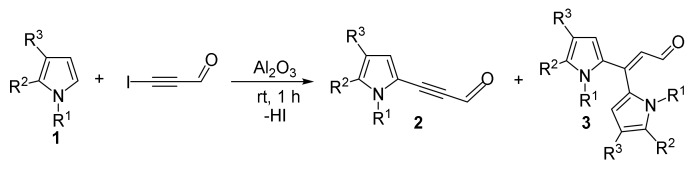
Reaction of pyrroles **1** with iodopropiolaldehyde in solid Al_2_O_3_.

In this case, the use of Al_2_O_3_ proved to be inappropriate, since the above ethynylation, albeit accelerated (the reaction time was 1 h), proceeded non-selectively to form, along with the target 3-(pyrrol-2-yl)propiolaldehydes **2**, 3-bis(pyrrol-2-yl)acrylaldehydes **3**, with the molar ratio being ~1:1 (Scheme 2).

It was found that 2-phenylpyrrole (**1**, R^1^ = H, R^2^ = Ph, R^3^ = H) gave the lowest yield (25%) of the ethynylated product that is likely associated with side reactions of the NH-function, e.g., nucleophilic addition across the triple bond or condensation with the aldehyde moiety.

**Scheme 3:**
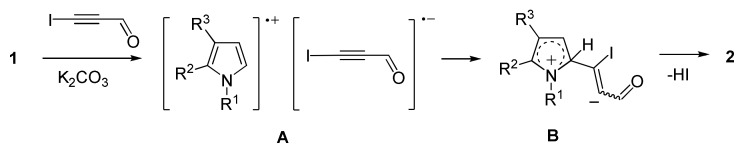
Reaction of pyrroles**1** with iodopropiolaldehyde in solid Al_2_O_3_.

The mechanism of the cross-coupling involves the single-electron transfer (SET) from pyrrole to iodopropiolaldehyde to generate the radical-ion pair A and/or the formation of the zwitterion B followed by elimination of hydrogen iodide (Scheme 3). Apparently, the role of K_2_CO_3_ is to stabilize the intermediate ion pairs by dipole-dipole interaction inside the ionic crystalline lattice of the medium, thus somewhat resembling ionic liquids.

The generation of radical-ions during this process was evidenced from ESR signals observed in the reaction of 1-vinyl-2-phenyl-3-amylpyrrole (**1**, R^1^ = CH=CH_2_, R^2^ = Ph, R^3^ = C_5_H_11_) with iodopropiolaldehyde in solid K_2_CO_3_. 

#### 2.1.2. Cross-Coupling of Acylbromoacetylenes with Pyrroles 

##### With 2-(Furan-2-yl)- and 2-(2-Thiophen-2-yl)pyrroles

**Scheme 4:**
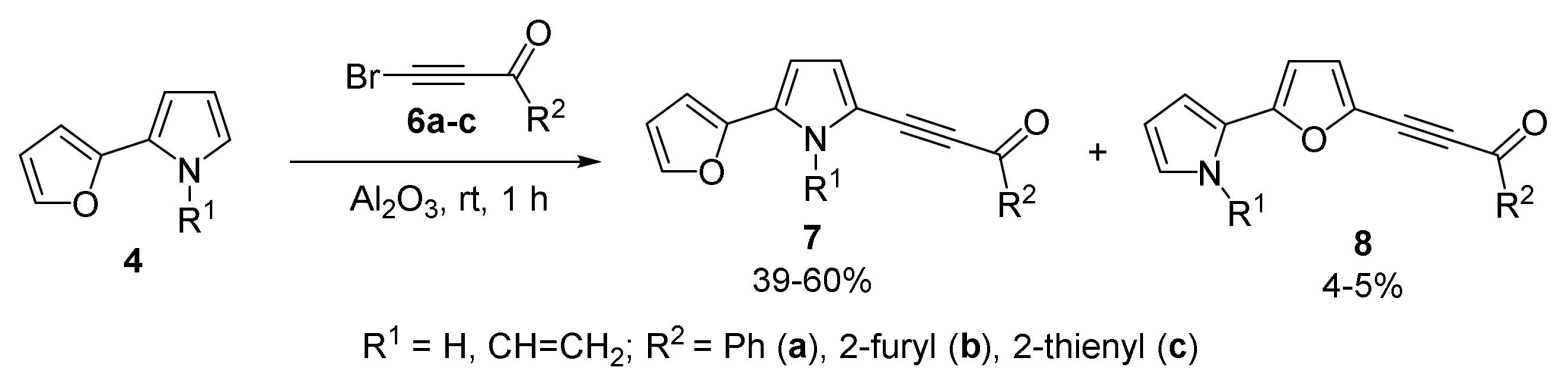
Reaction of 2-(furan-2-yl)pyrroles **4** with acylbromoacetylenes **6a**–**c** in the solid Al_2_O_3_.

The reaction of 2-(furan-2-yl)- (**4**) and 2-(thiophen-2-yl)pyrroles **5** with acylbromoacetylenes **6a**–**c** was carried out according to the similar procedure: the reactants (1:1 molar ratio) were ground with a 10-fold excess of Al_2_O_3_ at room temperature for 1 h [54]. The major direction of this ethynylation of 2-(furan-2-yl)pyrroles **4** was the formation of 2-acylethynyl-5-(furan-2-yl)pyrroles **7** (Scheme 4), while the alternative 2-acylethynyl-5-(pyrrol-2-yl)furans **8** were minor products (**7**:**8** = ~5–7:1). This result was key to understanding the ethynylation of five-membered aromatic heterocycles with haloacetylenes. In fact, this was the first observation of a relative reactivity of the furan ring in this reaction.

Double ethynylation, i.e., ethynylation of each ring, was not observed in any cases. In other words, the reaction occurs either with the pyrrole or furan ring. This points to a strong deactivating effect of the acyl substituent that is transmitted from one ring to another through the system of ten bonds involving conjugated one triple, four double, and five ordinary bonds. The ratio of products **7**:**8** = ~5–7:1 can be considered as an approximate measure of relative reactivity of the pyrrole and the furan ring towards the acylhaloacetylenes. The reaction of pyrroles with electrophilic acetylenes is commonly regarded as a nucleophilic addition of electron-rich pyrrole moiety (often as the pyrrolate anion) to the electron-deficient triple bond which occurs as N- and C-vinylation [55]. As mentioned above (see Section 2.1.1.) this reaction is likely initiated by the single-electron transfer to generate the radical-ion pairs as key intermediates, further forming C-C covalent bond with a final elimination of hydrogen halide [33]. Such a mechanism and the experimental isomer ratios are in agreement with a lower ionization potential of the pyrrole ring (8.09 eV) compared to that of furan ring (8.69 eV) [56].

**Scheme 5:**
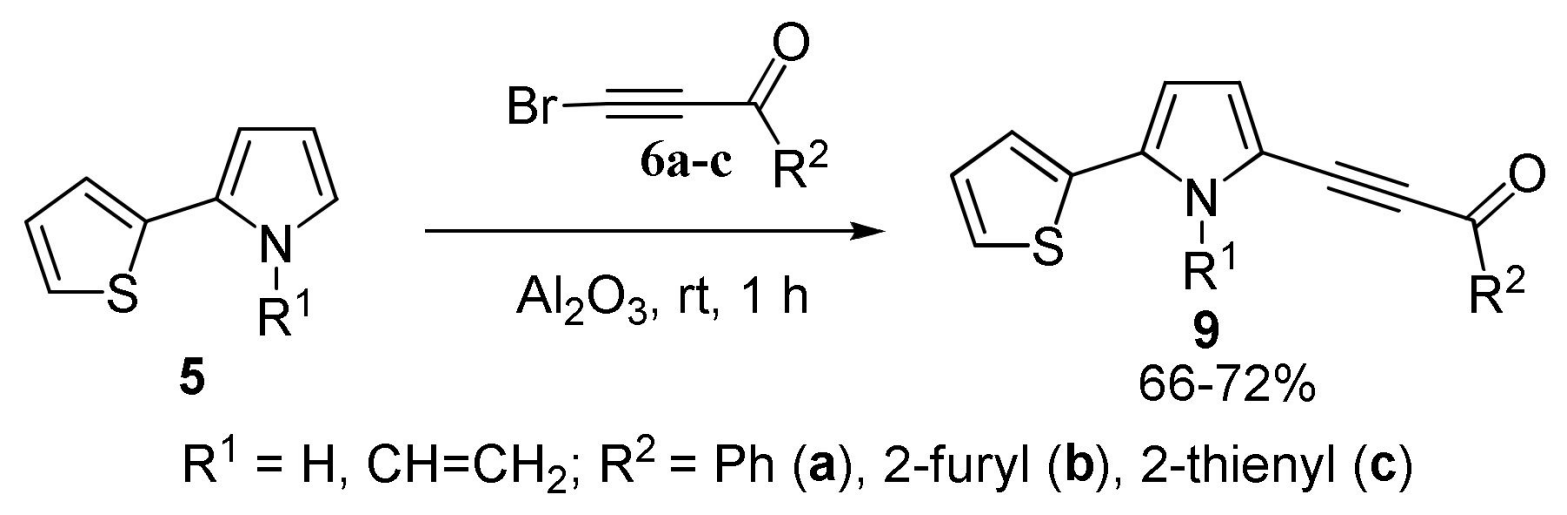
Reaction of 2-(thiophen-2-yl)pyrroles **5** with acylbromoacetylenes **6a**–**c** in the solid Al_2_O_3_.

In accordance with this rationale, 2-(thiophen-2-yl)pyrroles **5** reacted with acylbromoacetylenes **6a**–**c** under the above conditions to give only products of the pyrrole ring ethynylation, ethynylpyrroles **9** (Scheme 5) that also agrees with a higher ionization potentials of the thiophene ring (8.72 eV) [56].

**Scheme 6:**
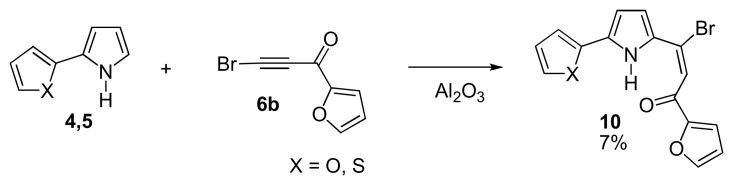
Formation of (*E*)-3-bromo-1-(pyrrol-2-yl)prop-2-en-1-ones **10**.

In cases of NH-pyrroles (**4**, **5**, R^1^ = H), 3-bromo-1-(pyrrol-2-yl)prop-2-en-1-ones **10** were isolated as the *E*-isomers stabilized by a strong intramolecular hydrogen bond between NH-proton and oxygen atom of the carbonyl group (Scheme 6).

The similar propenones were not observed among the products of ethynylation of N-vinylpyrroles (**4**, **5**, R^1^ = CH=CH_2_) because they are not able to form the above stabilizing intramolecular hydrogen bonding.

##### With Dipyrromethanes

**Scheme 7:**
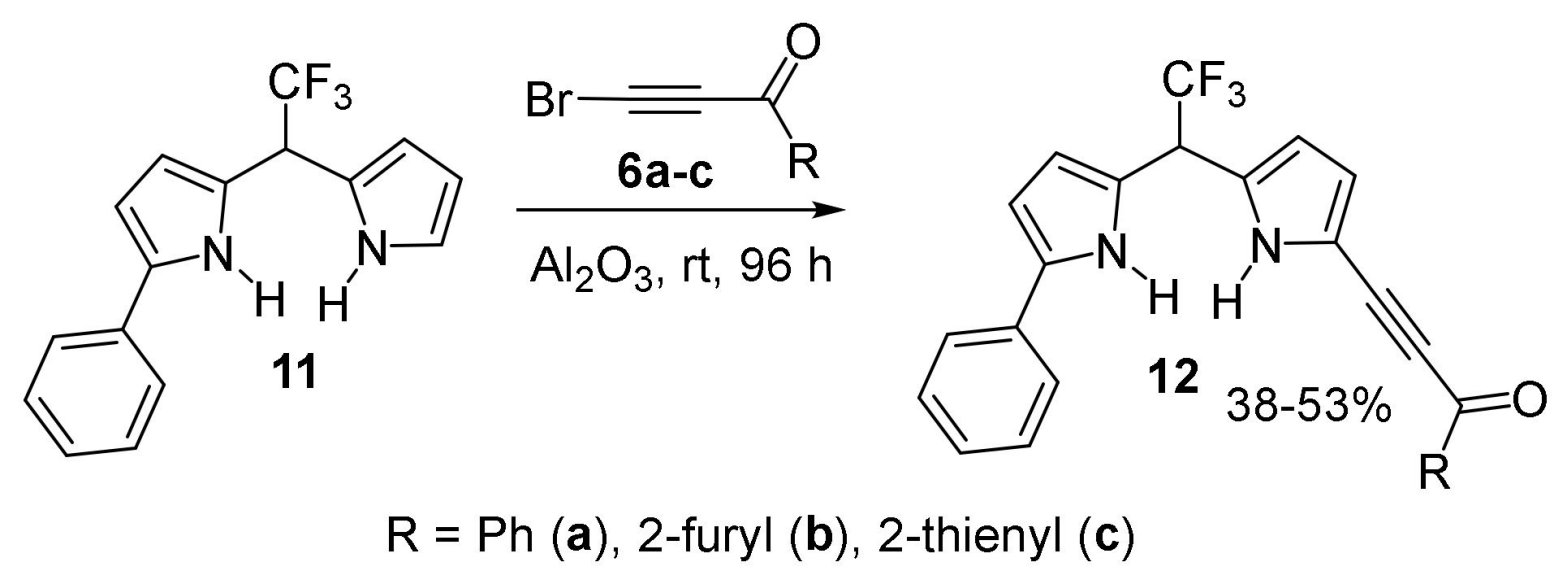
Ethynylation of the dipyrromethane **11** in the solid Al_2_O_3_.

The solid-phase (Al_2_O_3_) ethynylation of dipyrromethane **11** with acylbromoacetylenes **6a**–**c** afforded 5-acylethynyldipyrromethanes **12** in 38–53% yields (Scheme 7) [57]. In contrast to the ethynylation of pyrrole giving 2-acylethynylpyrroles in the yield of 55–70% for 1 h [20], the cross-coupling of dipyrromethane **11** with acylbromoacetylenes **6a**–**c** required a much longer time and portion-wise addition of acetylene **6a**–**c** to the reaction mixture.

The low reaction rate in this case is likely resulted from the strong electron-withdrawing effect of the CF_3_-group, deactivating the pyrrole ring that acts as a nucleophile. 

A general synthesis of such non-symmetrical dipyrromethanes was previously developed [58] by the condensation of trifluoropyrrolylethanols with pyrrole. 

In the solid K_2_CO_3_, effective in the ethynylation of pyrroles with haloacetylenes [35], the above reaction did not take place at all. 

**Scheme 8:**
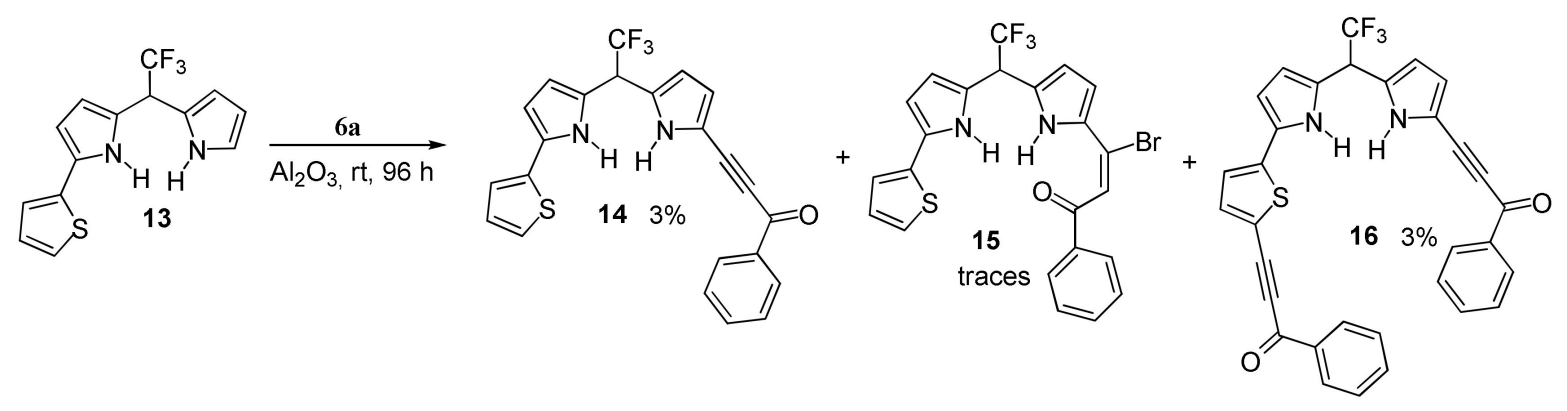
Ethynylation of the dipyrromethane **13** in the solid Al_2_O_3_.

In the solid alumina (room temperature, 96 h), dipyrromethane **13** reacted with benzoylbromoacetylene **6a** to give insignificant amounts of products. From the reaction mixture, apart from the target dipyrromethane **14**, 5-(1-bromo-2-benzoylethenyl)dipyrromethane **15**, and the double ethynylation product, (dibenzoylethynyl)dipyrromethane **16**, were isolated in low yields (Scheme 8). The formation of dipyrromethane **16** was the first example of ethynylation of the thiophene ring by the reaction studied.

**Scheme 9:**
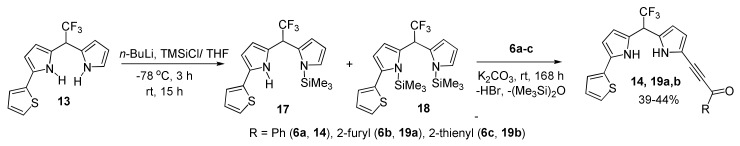
Synthesis and ethynylation of dipyrromethanes **17**, **18**.

To increase the nucleophilicity of the pyrrole ring, trimethylsilyl group was introduced to nitrogen atom of the pyrrole ring (Scheme 9). The ethynylation (K_2_CO_3_, room temperature, 168 h) of a mixture of dipyrromethanes **17** and **18** with acylbromoacetylenes **6a**–**c** gave acylethynyldipyrromethanes **14, 19** in 39–44% yields (Scheme 9). Thus, the yields of ethynylated product were increased due to the introduction of trimethylsilyl groups in the pyrrole ring to enhance their nucleophilicity.

##### With Tetrahydropyrrolo [3,2-*c*]pyridines

**Scheme 10:**
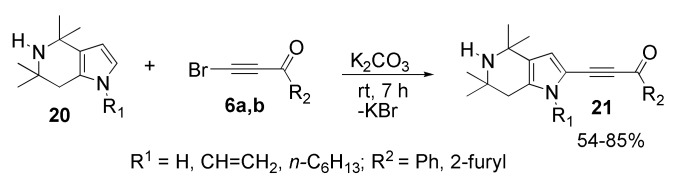
Cross-coupling of tetrahydropyrrolo[3,2-*c*]pyridines **20** with acylbromoacetylenes **6a**,**b** in the solid K_2_CO_3_.

The cross-coupling of pyrrolo[3,2-*c*]pyridines **20** with acylbromoacetylenes **6a**,**b** in solid K_2_CO_3_ was strictly chemo- and regioselective: exclusively propynones **21** were isolated (Scheme 10) [59].

In this case, the use of K_2_CO_3_ appeared to be essential, since it allowed the released HBr to be effectively fixed. This prevented the salt formation with the NH-function of the tetrahydropyridine moiety.

**Scheme 11:**
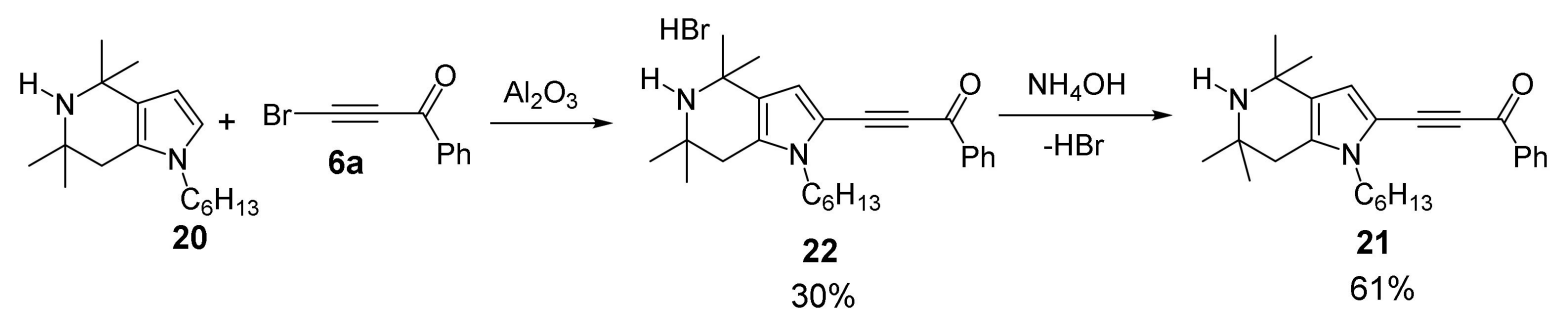
Cross-coupling of pyrrolo[3.2-*c*]pyridine **20** with benzoylbromoacetylene **6a** in the solid Al_2_O_3_.

Indeed, when Al_2_O_3_ (instead of K_2_CO_3_) served as an active medium, the reaction of pyrrole **20** (R^1^ = C_6_H_13_) with benzoylbromoacetylene **6a** afforded salt of propynone, hydrobromide **22** (Scheme 11). Upon treatment of the aqueous solution of salt **22** with NH_4_OH propynone **21** was obtained in 61% yield.

##### With Pyrrole-2-carbaldehydes

**Scheme 12:**
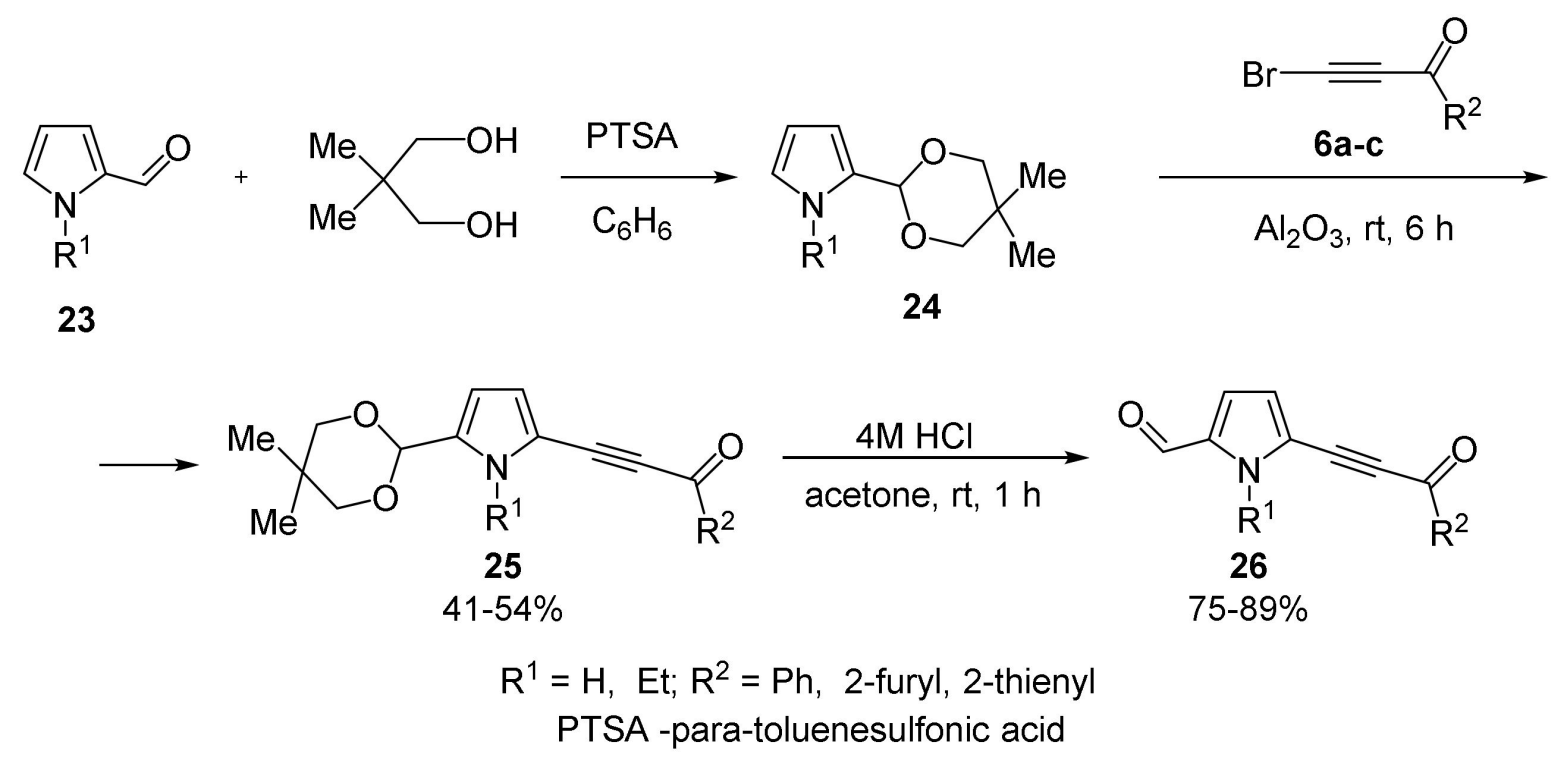
Synthesis of pyrrole-2-carbaldehydes with the electron-deficient acetylenic substituents.

Pyrrole-2-carbaldehydes **23** proved to be inactive under usual conditions of the cross-coupling of pyrroles with acylhaloacetylenes in alumina medium (room temperature, 1 h). The reason is likely strong electron-withdrawing effect of the aldehyde group which decreases the pyrrole ring nucleophilicity. This fundamental hurdle was overcome by the acetal protection of the aldehyde function thereby decreasing its electron-withdrawing power [60,61]. The acetals **24** were treated with acylbromoacetylenes **6a**–**c** in the alumina medium (room temperature, 6 h) to obtain the expected ethynylated acetals **25**. After the deprotection (aqueous acetone, HCl, room temperature, 1 h), the target ethynylated pyrrole-2-carbaldehydes **26** were isolated in 75–89% yields (Scheme 12).

#### 2.1.3. Cross-Coupling of Bromotrifluoroacetylacetylene with Pyrroles

**Scheme 13:**
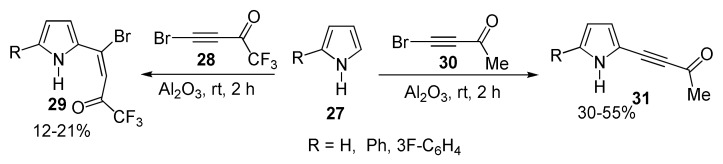
The cross-coupling of NH-pyrroles **27** with acetylbromoacetylenes **28** and **30** in the solid Al_2_O_3_.

Pyrroles **27**, when reacted with bromotrifluoroacetylacetylene **28** in the solid Al_2_O_3_ (room temperature, 2 h), gave only 4-bromo-1,1,1-trifluoro-4-(pyrrol-2-yl)but-3-en-2-ones **29** in 12–21% yields [62,63], while the cross-coupling of acetylbromoacetylene **30** with the same pyrroles under the same conditions afforded the expected acetylethynylpyrroles **31** (Scheme 13) [62].

**Scheme 14:**
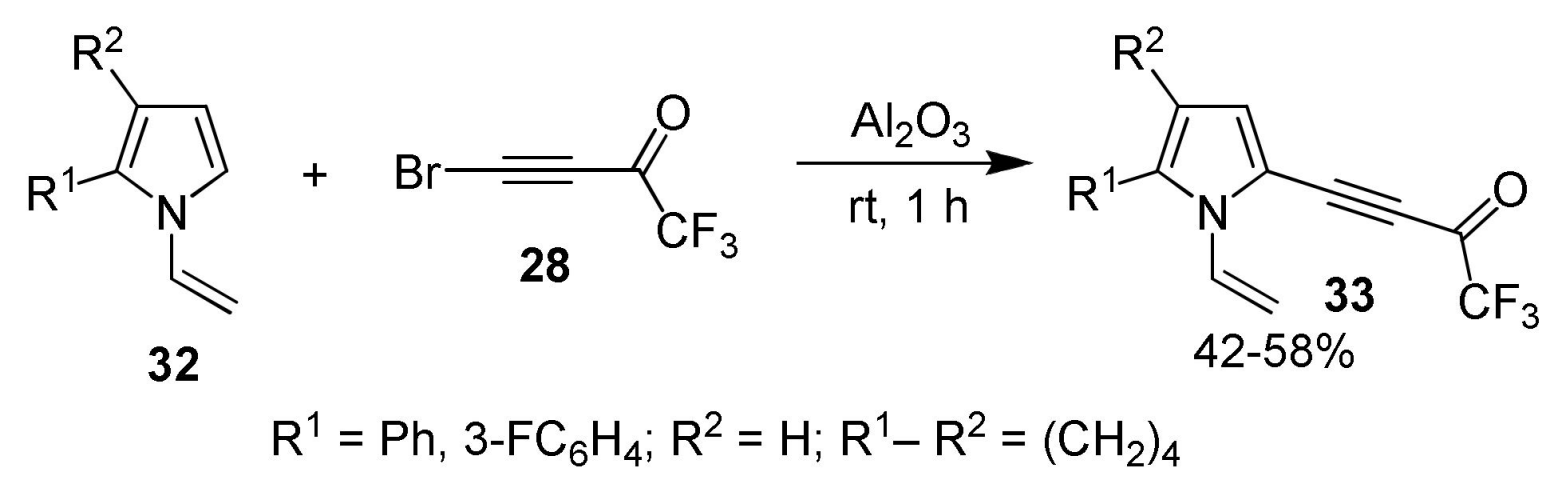
The cross-coupling of *N*-vinylpyrroles **32** with bromotrifluoroacetylacetylene **28** in the solid Al_2_O_3_.

Interestingly, *N*-vinylpyrroles **32** underwent normal cross-coupling with bromotrifluoroacetylacetylene **28** (Al_2_O_3_, rt, 2 h) to deliver ethynylpyrroles **33** in 42–58% yields (Scheme 14).

This implies that the cause of abnormal reaction (Scheme 13) is the interaction between NH and trifluoroacetyl groups that stabilizes 4-bromo-1,1,1-trifluoro-4-(pyrrol-2-yl)but-3-en-2-ones **29** in their *E-*configuration.

This is evidenced from the extraordinary downfield shift of the NH group proton signal (13–14 ppm) in the ^1^H NMR spectra of pyrroles **29**.

**Scheme 15:**
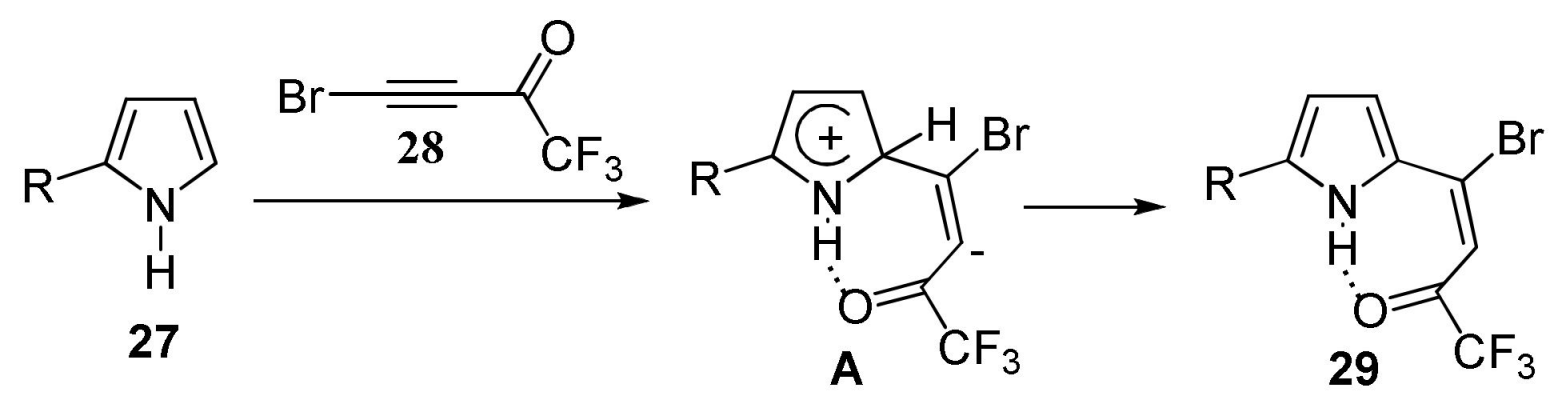
Proposed mechanism of formation of *E*-isomers of the hydrogen-bonded compounds **29**.

The intramolecular H-O-bonding of such a type is likely realized already in the *E*-form of the intermediate zwitterion **A** (Scheme 15). This hydrogen bonding prevents the *E*↔*Z* isomerization and hence elimination of HBr, which usually occurs as a *trans*-process. Notably, in most cases of ethynylation of pyrroles under similar conditions [20], bromopyrrolylethenylketones of the type **29** are formed just as minor contaminants, if any (0–10% yields), that may also be a result of easier elimination of hydrogen halides (HBr in this case) from their *Z*-configuration. As the elimination of HBr does not occur at a stage of the zwitterion **A** formation, the proton in the 2 position of the pyrrole ring is transferred to the carbanionic center. This should be facilitated by a strong electron-withdrawing effect of trifluoroacetyl substituent. Consequently, the target product **29** is formed stereoselectively (as the *E*-isomer).

**Scheme 16:**
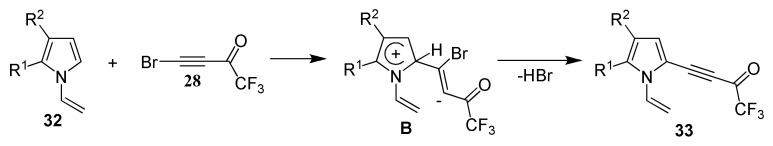
Formation of products **33** from N-vinylpyrroles **32** and bromotrifluoroacetylacetylene **28**.

In the reaction of *N*-vinylpyrroles **32** with the bromotrifluoroacetylacetylene **28**, the formation of an intramolecular hydrogen bond in the products is impossible (Scheme 16). Moreover, the formation of the *E*-isomer of 4-bromo-1,1,1-trifluoro-4-(pyrrol-2-yl)but-3-en-2-ones **B** would be sterically hindered (due to the repulsion between N-vinyl group and trifluoroacetyl substituent).

Probably, the effect of steric strain destabilizes the *E*-form at the stage of formation of the intermediate zwitterion **B** (Scheme 16), for which the Z-form turns out to be energetically favorable. At the final stage, the zwitter-ion **B** is transformed to trifluoroacetylethynylpyrroles **33** via elimination of the bromine anion accompanied by releasing of proton from the position 2 of the pyrrole ring (Scheme 16).

**Scheme 17:**
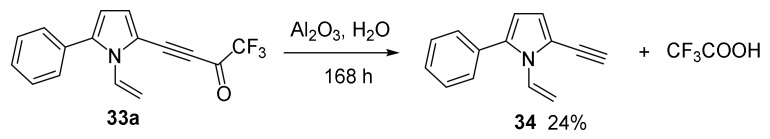
Detrifluoroacylation of compound **33a** after 7 days contact with Al_2_O_3_.

Pyrrole **33a**, after 7 days contact with Al_2_O_3_, lost the trifluoroacetyl group to give 2-ethynylpyrrole **34** in 24% yield (Scheme 17). The partial detrifluoroacylation of pyrroles **33** also occurred during their passing through Al_2_O_3_-packed chromatographic column.

#### 2.1.4. Cross-Coupling of Chloroethynylphosponates with Pyrroles

**Scheme 18:**
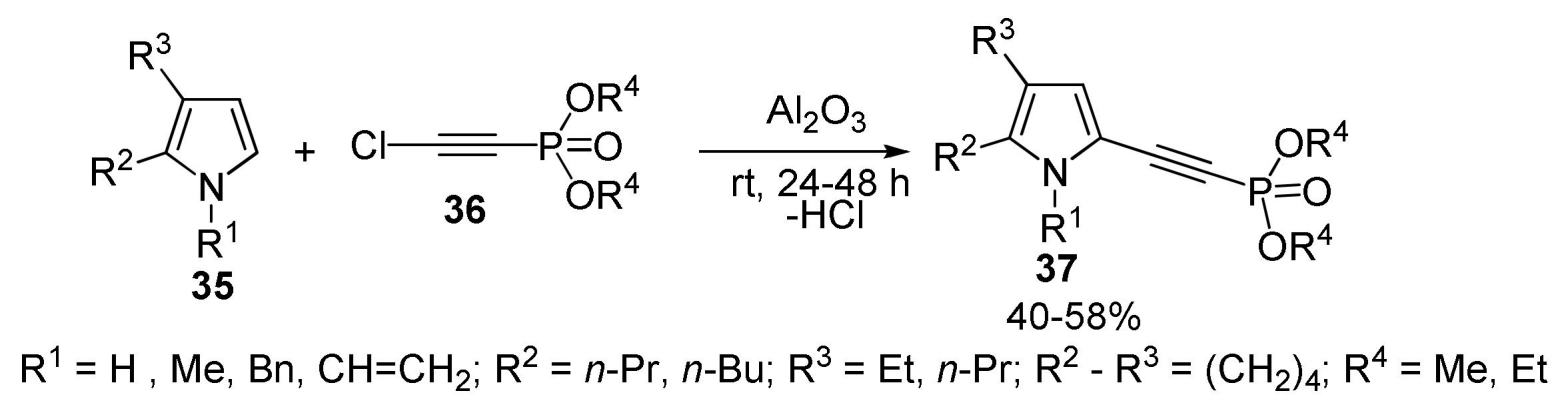
Synthesis of 2-(pyrrol-2-yl)ethynylphosphonates **37**.

Pyrroles **35** were cross-coupled with chloroethynylphosponates **36** in solid alumina (room temperature, 24–48 h) to give 2-(pyrrol-2-yl)ethynylphosphonates **37** in 40–58% yields (Scheme 18) [64].

In the absence of Al_2_O_3_ (both in a solvent and under solvent-free conditions), the ethynylation did not take place. At room temperature, the complete conversion of the reactants was reached after 24 h. The exception was *N*-vinyl-4,5,6,7-tetrahydroindole **35a** (R^1^ = H; R^2^-R^3^ = (CH_2_)_4_), the ethynylation of which lasted twice as long (48 h).

**Figure 1:**
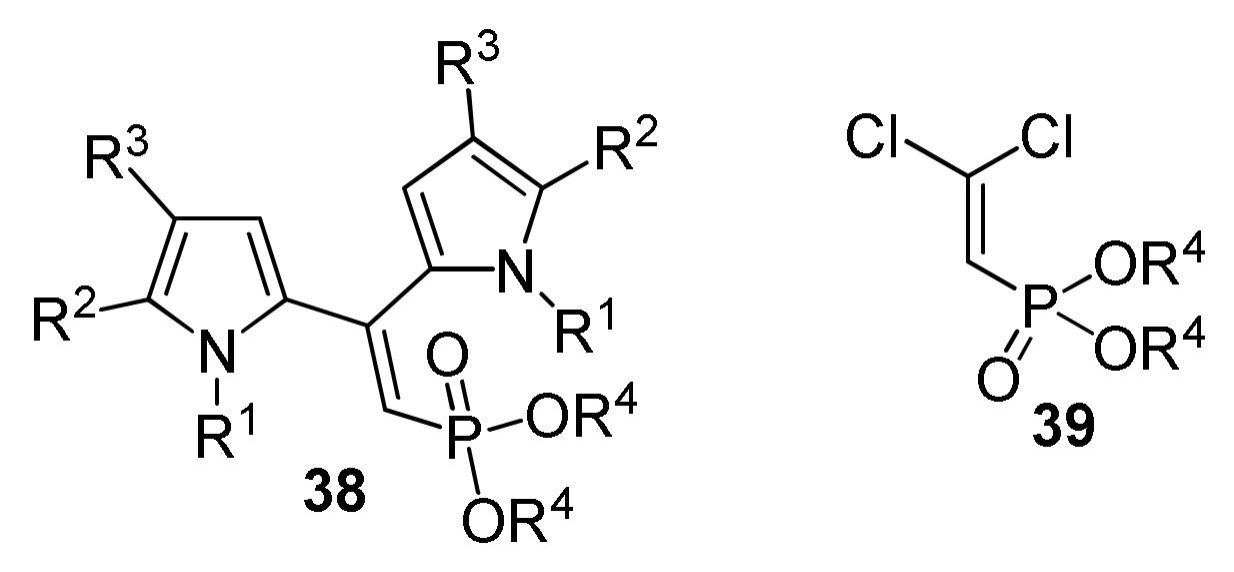
Side products of cross-coupling of pyrroles **35** with chloroethynylphosphonates **36**.

As minor products (2–10%), 2,2-*bis*(pyrrol-2-yl)vinylphosphonates **38** were detected in the reaction (Figure 1). Also, in the case of 1-vinyl-4,5,6,7-tetrahydroindole [**35**, R^1^ = CH=CH_2_; R^2^-R^3^ = (CH_2_)_4_], dialkyl 2,2-dichlorovinylphosphonates **39** (2–9%) were present in the reaction mixture (Figure 1).

The formation of the product **39** required [64] a longer reaction time (48 vs. 24 h) that allowed hydrogen chloride to be competitively added to the starting chloroethynylphosphonates according to [65]. A longer reaction rate is also due to the electron-withdrawing effect of the vinyl group, which reduces nucleophilicity of the pyrrole moiety.

In the solid K_2_CO_3_ medium (other conditions being the same), the cross-coupling of pyrroles with chloroethynylphosphonates produced only pyrrolylethynylphosphonates **37** in 38–43% yields.

It is suggested [64] that the reaction mechanism in this case represents the direct nucleophilic substitution of chlorine atom by the pyrrole moiety. This is supported by known data [66,67] that the reactions of chloroethynylphosphonates with nucleophiles including the neutral ones proceed mainly as a nucleophilic substitution of chlorine atom at the C_sp_ carbon.

#### 2.1.5. Cross-Coupling of Halopolyynes with Pyrroles

The above transition metal-free solid-phase mediated cross-coupling of haloacetylenes with pyrroles turns out to be efficient also for halopolyynes (di-, tri-, and tetrapolyynes) [68,69], allowing the pyrroles functionalized with polyynes chains to be synthesized. Such rare, highly reactive pyrrole compounds represent exclusively promising building blocks and precursors for the design of biologically and technically valuable heterocyclic molecules of exceptional complexity and structural diversity, including porphyrinoids with the polyyne substituents [70], modified bilirubins [71], and various ensembles of pyrroles with furans [72,73], thiophenes [72,73], pyrroles [72,73], naphthalenes [73], and other cyclic counterparts [73].

**Scheme 19:**
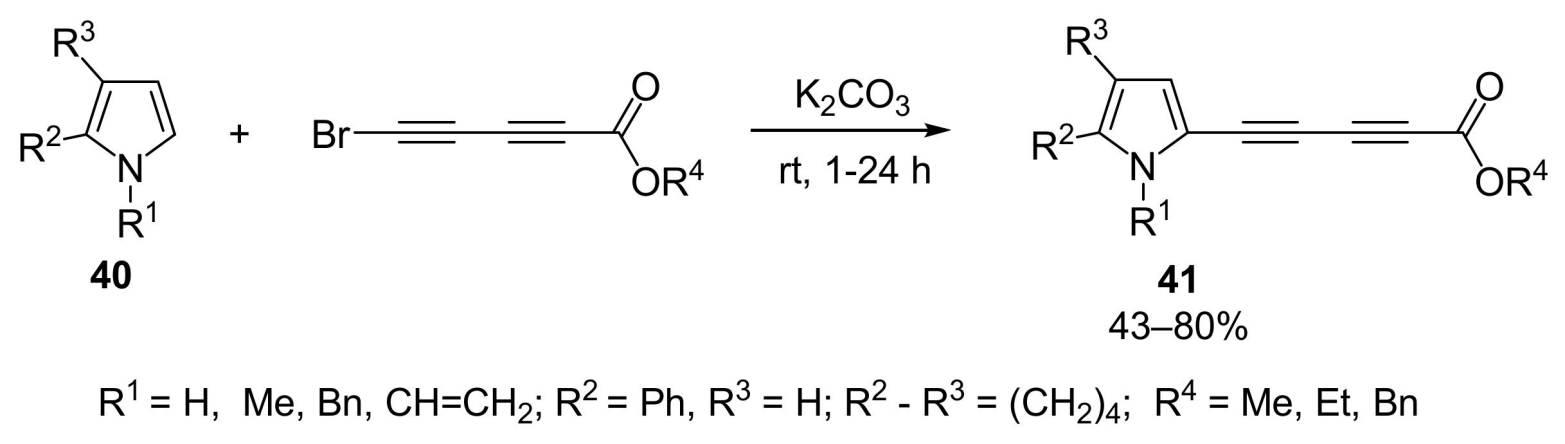
Cross-coupling of electron-deficient halobutadiynes with pyrroles **40**.

Thus, ester end-capped 1-halobutadiynes were successfully cross-coupled with pyrroles **40** in the solid K_2_CO_3_ to afford the expected butadiynyl-substituted pyrroles **41** in 43–80% yields (Scheme 19) [68].

The scope of reactions covers 2-phenylpyrrole, NH-4,5,6,7-tetrahydroindole, N-substituted 4,5,6,7-tetrahydroindoles, and chloro-, bromo-, and iodobutadiynes. The most suitable butadiynyl agents proved to be 1-bromobutadiynes.

The reaction rate depends on the pyrrole structure, with tetrahydroindole derivatives being the most reactive. For them, the cross-coupling with one equivalent of various halobutadiynes did not exceed 5 h, whereas for 2-phenylpyrrole, to reach 46–52% yield of the target product it required 2 equivalents of halobutadiynes and much longer reaction time (24 h).

This study was further extended over the longer chain aryl-capped 1-halopolyynes (up to tetraynes) [69]. As pyrrole substrates, 4,5,6,7-tetrahydroindole and its N-substituted derivatives were employed.

**Scheme 20:**
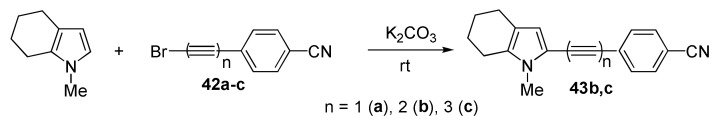
The reaction of polyynes **42a**–**c** with *N*-methyl-4,5,6,7-tetrahydroindole.

For the interaction of *N*-methyl-4,5,6,7-tetrahydroindole with 1-bromo-2-(4-cyanophenyl)acetylene **42a**, 1-bromo-2-(4-cyanophenyl)butadiyne **42b**, 1-bromo-2-(4-cyanophenyl)hexatriyne **42c**, the expected cross-coupling was observed only for triyne **42c** (K_2_CO_3_, room temperature, 3 h, 82% yield of hexatriynyl substituted *N*-methyl-4,5,6,7-tetrahydroindole **43c**), while with acetylene **42a** no target product was detected (^1^H-NMR), and in the case of diyne **42b** a slow reaction (several days) took place (Scheme 20).

**Scheme 21:**
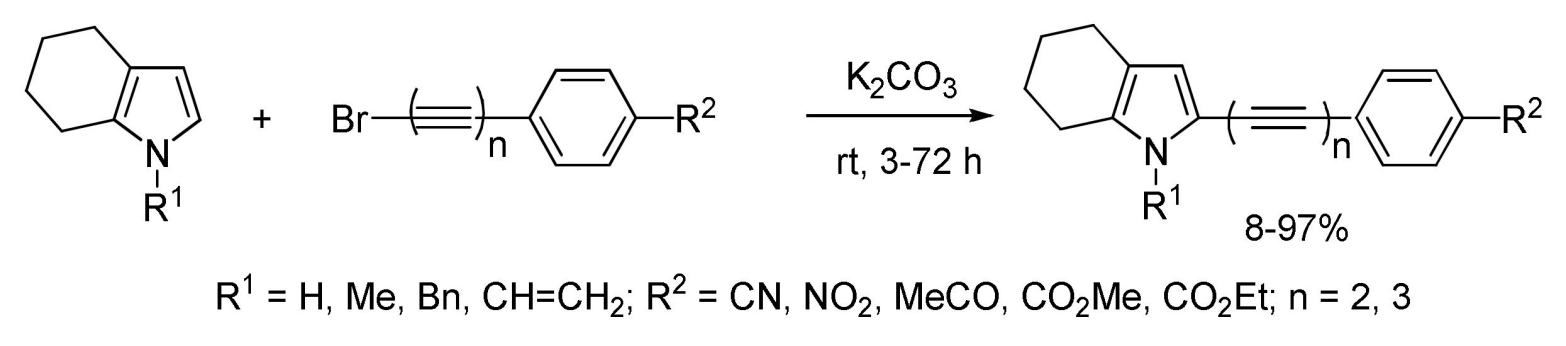
Synthesis of tetradiynyl- and hexatriynyl-substituted tetrahydroindoles.

**Scheme 22:**
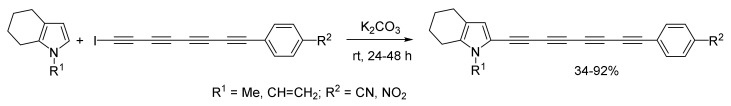
Synthesis of octatetraynyl-substituted tetrahydroindoles.

Since longer bromopolyynes were less stable than the corresponding iodine derivatives, 1-bromotetra- and -hexatriynes were used for the synthesis of tetradiynyl- and hexatriynyl-substituted tetrahydroindoles (Scheme 21), while 1-iodotetraynes were employed to produce octatetraynyltetrahydroindoles (Scheme 22).

**Scheme 23:**
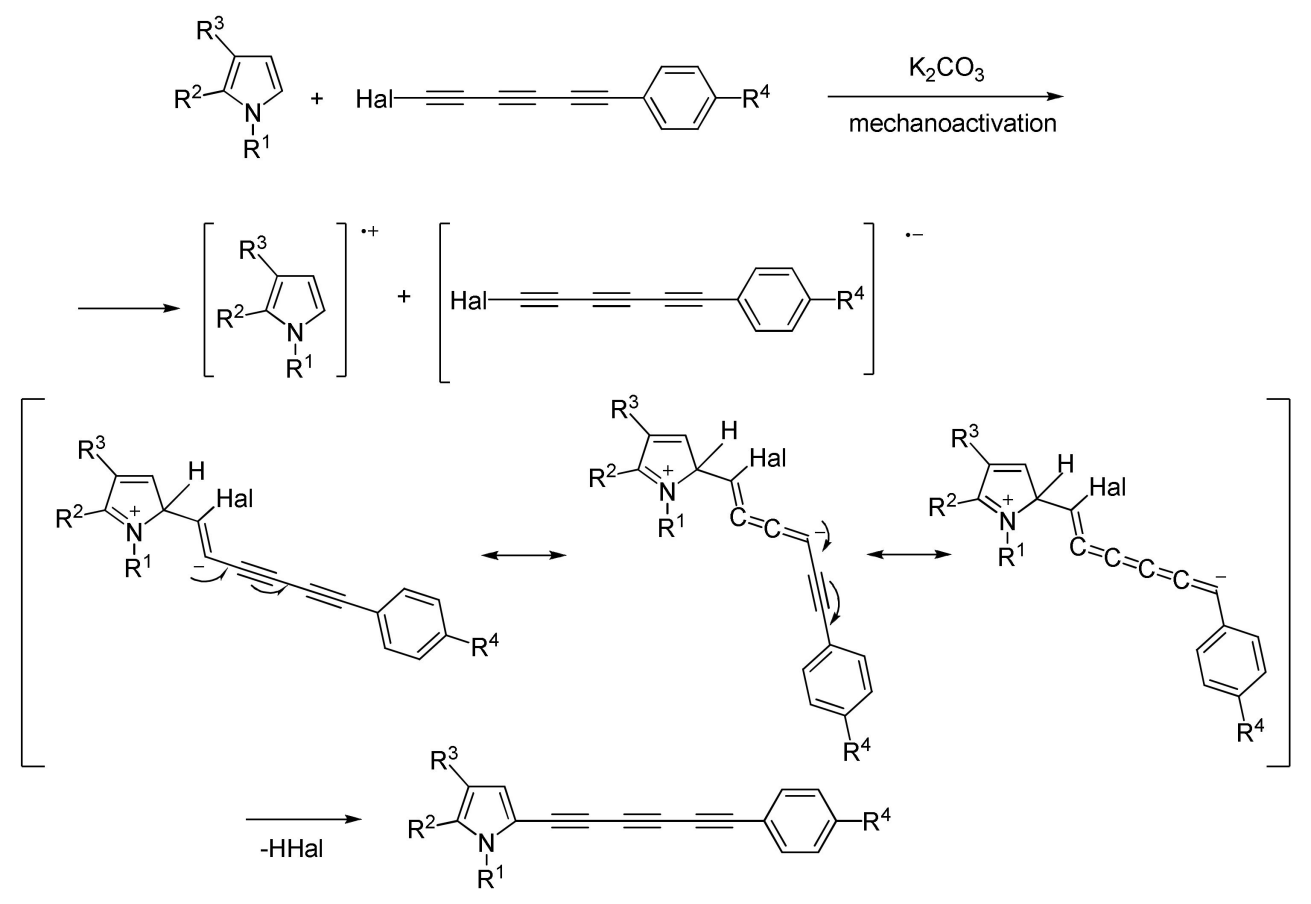
Proposed mechanism of long-chain stabilization of a radical intermediate product.

Like in the work of Trofimov B.A. [33] (see also Section 2.1.1.), it is assumed that the reaction mechanism involves radical-ion pairs generated by the SET process (Scheme 23). According to experimental results longer polyyne chains secure a better stabilization of radical-ion pairs that provide higher yields of polyynyl substituted tetrahydroindoles and a shorter reaction time.

## 3. Reaction of Acylhaloacetylenes with Furans

A logical development of ethynylation of pyrroles with haloacetylenes [20] was the translation of this methodology to the furan compounds. In this line, on the example of menthofuran (3,6-dimethyl-4,5,6,7-tetrahydrobenzofuran **44**), first synthetically appropriate results on the transition metal-free cross-coupling of the furan ring with haloacetylenes **6a**–**f** initiated by their grinding with solid Al_2_O_3_ (room temperature, 1–72 h) were attained [74].

**Scheme 24:**
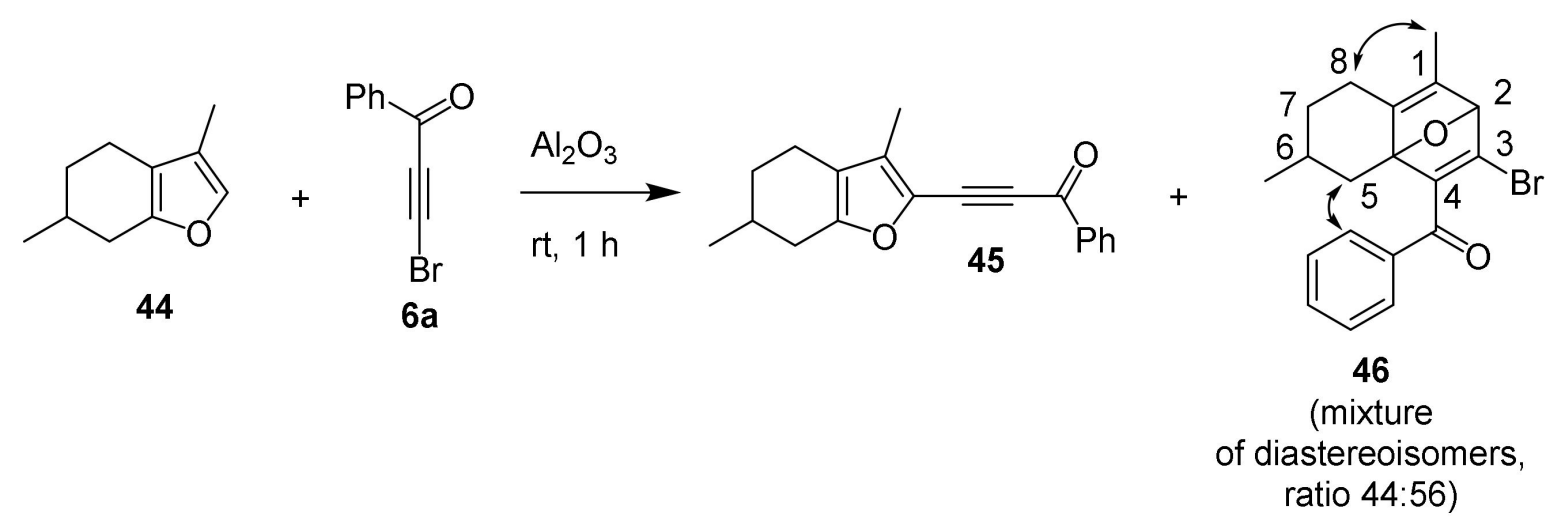
Reaction of benzoylbromoacetylene **6a** with menthofuran **44** in the solid Al_2_O_3_.

As it was found by Trofimov B.A. [74], after 1 h the reaction of menthofuran **44** with benzoylbromoacetylene **6a** in solid Al_2_O_3_ resulted in the formation of ethynylfuran **45** along with the pair of diastereomeric cycloadducts of oxanorbornadiene structure **46** in 44:56 ratio (Scheme 24). The reaction is regioselective: the bromine atom is neighboring the position 2 of the furan ring exclusively.

Upon standing the reaction mixture for 72 h, the content of ethynylfuran **45** was increased up to 88%, while amount of cycloadduct **46** was reduced. These results indicate that cycloadduct **46** converts to ethynylfuran **45**, i.e., the cycloadduct **46** is a kinetic intermediate of the ethynylation (this transformation is accompanied by elimination of hydrogen bromide).

The reaction of menthofuran **44** with chloro- and iodobenzoylacetylenes proceeded analogously leading for 1 h to a mixture of ethynylfuran **45** and cycloadduct **46**, the latter disappearing completely after 72 h.

Thus, in contrast to cross-coupling of pyrroles with acylhaloacetylenes under similar conditions, ethynylation of the furan ring with acylhaloacetylenes occurred through [4+2]-cycloaddition followed by the elimination of HX during the ring-opening of the cycloadducts.

**Scheme 25:**
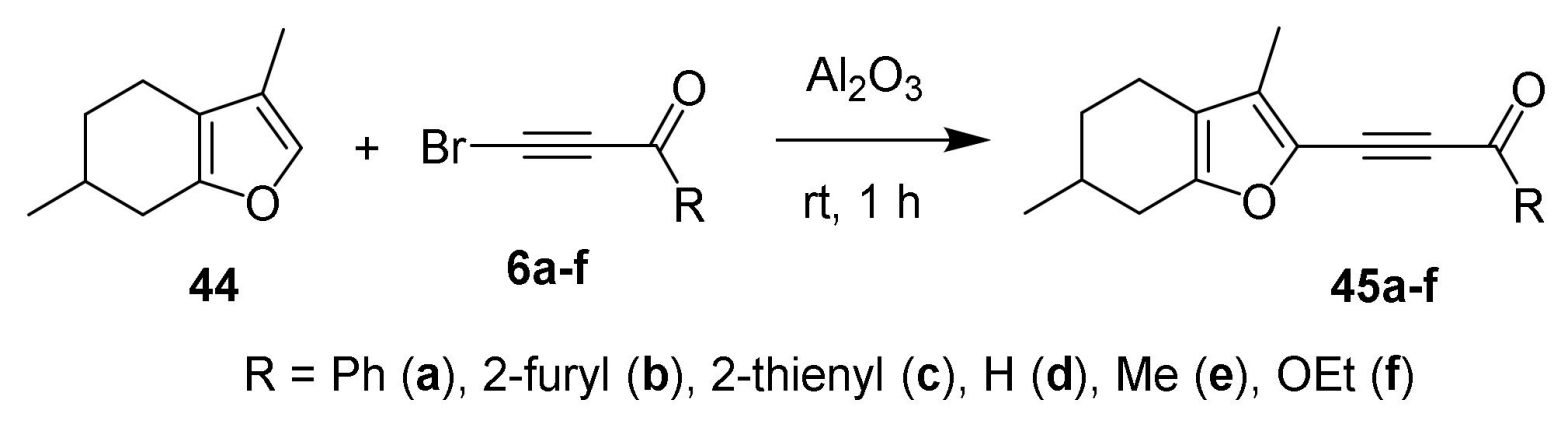
Reaction of haloacetylenes **6a**–**f** with menthofuran **44** in thesolid Al_2_O_3_.

This reaction was proved to be applicable to bromoacetylenes with formyl (**6d**), acetyl (**6e**), furoyl (**6b**), thenoyl (**6c**), and ethoxy (**6f)** groups at the triple bond, which reacted with menthofuran **44** in the solid Al_2_O_3_ to afford the acetylenic derivatives **45a**–**f** in 40–88% yields (Scheme 25).

**Scheme 26:**
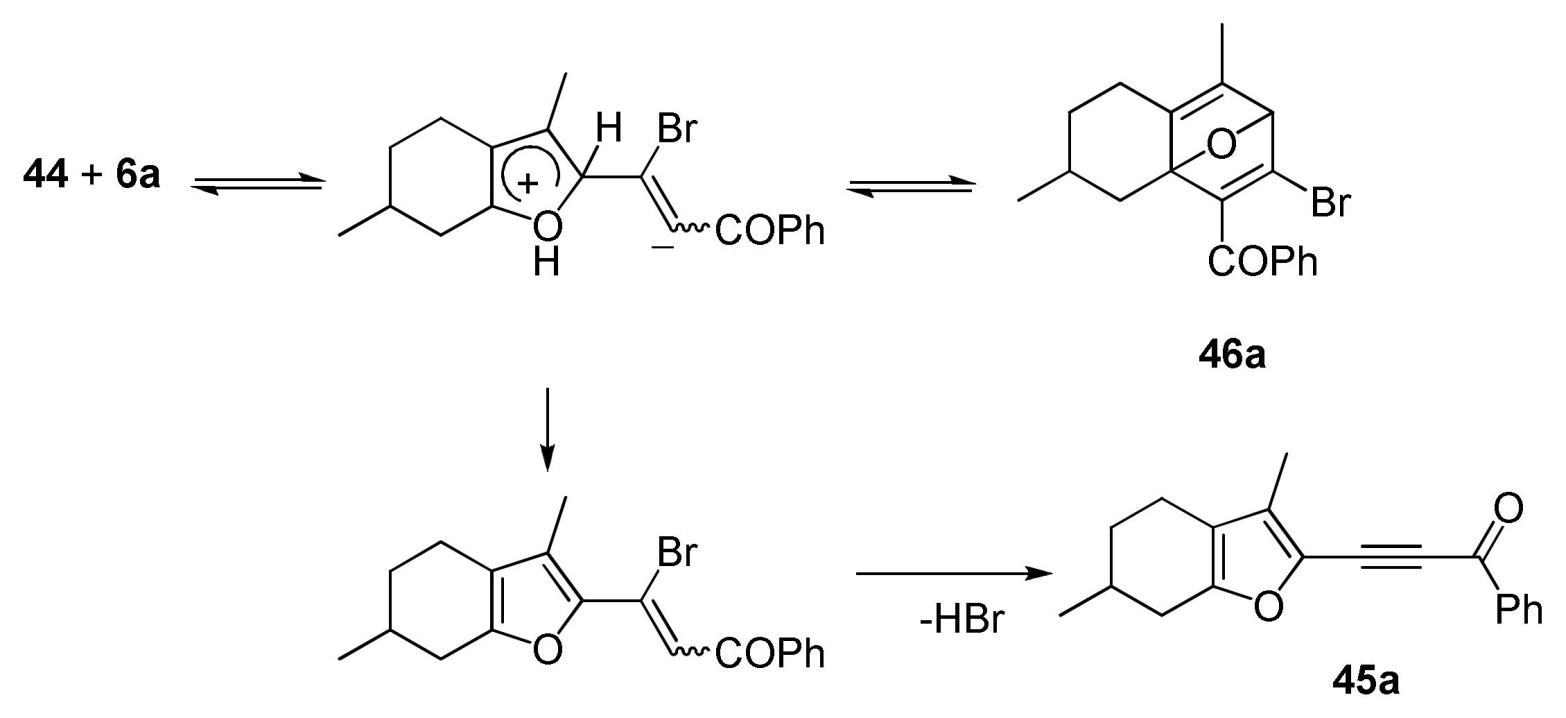
Possible reaction pathway.

Following the experimental results, the oxanorbornadiene intermediates such as **46**, reversibly generated on the first reaction step, are transformed to the ethynyl derivatives of menthofuran **45** via a zwitterion with the positive charge distributed over the whole furan ring. The latter eliminates hydrogen bromide in the concerted process (hydrogen is released from the position 2 of the furan moiety, Scheme 26).

An experimental evidence for the proposed mechanism is the observation that cycloadducts **46** are gradually transformed to ethynylated products **45** in the solid Al_2_O_3_.

## 4. Reaction of Acylhaloacetylenes with Pyrazoles

**Scheme 27:**
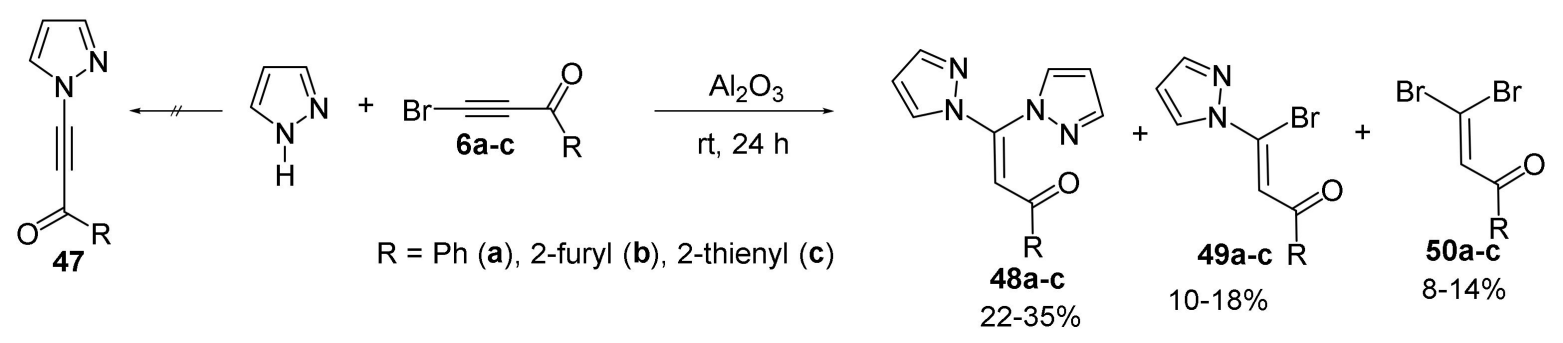
Reaction of pyrazole with acylbromoacetylenes **6a**–**c**.

The reaction of benzoylbromoacetylene **6a** with pyrazole under conditions similar for the ethynylation of pyrroles (10-fold excess of Al_2_O_3_, room temperature, the molar ratio 1:1, 24 h), instead of the expected 2-benzoylethynylpyrazole **47**, led to dipyrazolylenone **48a** in 18% isolated yield (Scheme 27) [75]. The yield of enone **48a** increased to 32%, when 2 equivalents of pyrazole was taken and reached 43% for the reaction with a 3-molar excess of the starting heterocycle.

The reaction proceeded via the intermediate (*Z*)-2-bromo-2-(pyrazol-1-yl)enone **49a** and was accompanied by the formation of 2,2-dibromoenone **50a** (Scheme 27).

Modest yields (22–35%) of dipyrazolylenones **48a**–**c** were observed using a two-fold molar excess of pyrazole relative to acylbromoacetylenes **6a**–**c**, with the yields of bromopyrazolylenones **49a**–**c** and dibromoenones **50a**–**c** being 10–18% and 8–14%, respectively (Scheme 27).

Surprisingly, no traces of ethynylpyrazoles **47** were detectable in the reaction mixture, implying that dipyrazolylenones **48a–c** are not adducts of the reaction of pyrazole with the intermediate ethynylated pyrazoles **47**.

**Scheme 28:**
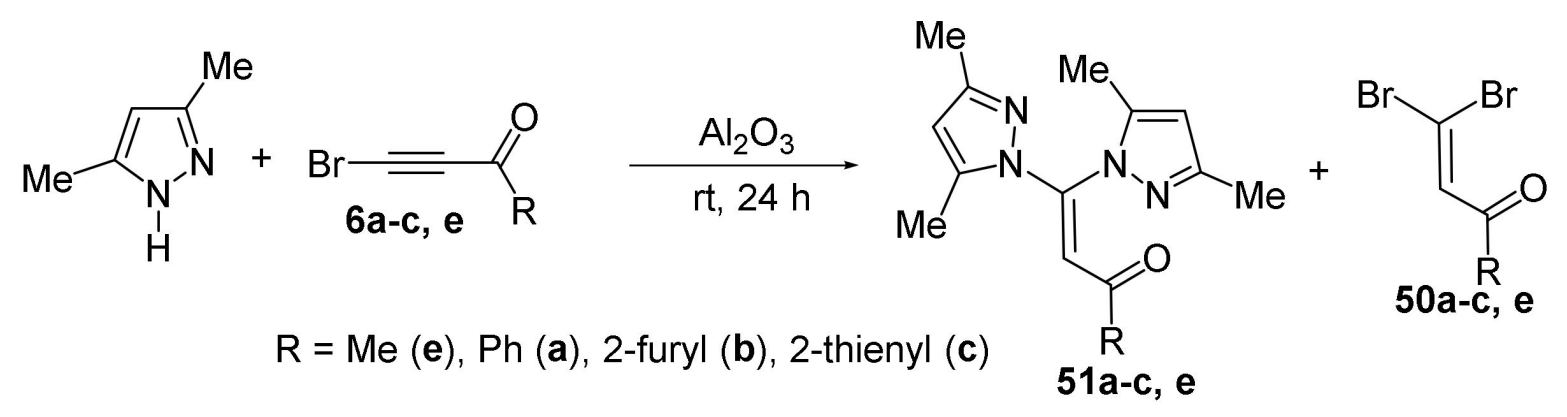
Reaction of 3,5-dimethylpyrazole with acylbromoacetylenes **6a**–**c, e**.

3,5-Dimethylpyrazole reacted with acylbromoacetylenes **6a**–**c**,**e** in a 2:1 molar ratio to form dipyrazolylenones **51a**–**c**,**e** in 42–55% yields (Scheme 28). Bromopyrazolylenone of the type **49** in this case, was not discernible in the reaction mixture.

**Scheme 29:**
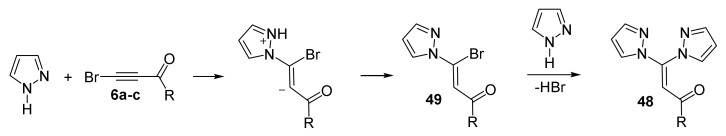
Proposed mechanism of dipyrazolylenones **48** formation.

On the basis of the results obtained and previous mechanistic rationalizations concerning the reactions of pyrroles with haloacetylenes [20], it may be suggested that the synthesis of dipyrazolylenones **48** is triggered by the nucleophilic addition of pyrazole to the triple bond of acylbromoacetylenes **6a**–**c** to form the intermediate zwitterion (Scheme 29), which converts via proton transfer from the pyrazole moiety to its carbanionic center to give isolable intermediate **49**. Subsequent nucleophilic substitution of the bromine atom by a second molecule of pyrazole affords dipyrazolylenone **48**.

Unlike the ethynylation of pyrroles, where the initial zwitterion releases a halogen anion to restore the triple bond, for pyrazole, rapid intramolecular neutralization of the carbanionic site of the intermediate zwitterion occurs, which precludes formation of the ethynyl derivatives. Such a change of the reaction mechanism is likely due to the higher acidity of pyrazoles compared with pyrroles (pk_a_ of pyrazole is 14.2 whereas pk_a_ of pyrrole is 17.5).

## 5. Selected Reactions of Acylethynylpyrroles and Their Analogs

To demonstrate the possibilities of the cross-coupling developed for the construction of important functionalized heterocyclic systems, some selected synthetically attractive reactions of acylethynylpyrroles and their analogs are considered below.

### 5.1. Cyclizations with Propargylamine

#### 5.1.1. Synthesis of Pyrrolo[1,2-*a*]pyrazines

**Scheme 30:**
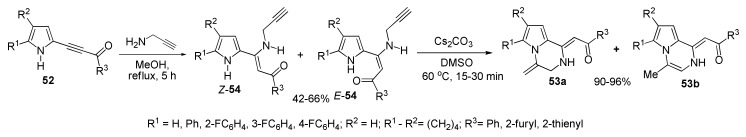
Synthesis of pyrrolo[1,2-*a*]pyrazines **53a,b** from acylethynylpyrroles **52** and propargylamine.

Acylethynylpyrroles **52** were used for the synthesis of pyrrolo[1,2-*a*]pyrazines **53a,b** according to the strategy which includes the following steps: (i) the non-catalyzed chemo- and regioselective nucleophilic addition of propargylamine to the triple bond of acylethynylpyrroles **52** to afford *N*-propargyl(pyrrolyl)aminoenones **54** and (ii) base-catalysed intramolecular cyclization of *N*-propargyl(pyrrolyl)aminoenones **54** to pyrrolo[1,2-*a*]pyrazines **53a,b** (Scheme 30) [76].

Nucleophilic addition of propargylamine to the triple bond of acylethynylpyrroles **52** was carried out under reflux of reactants (**52**: propargylamine ratio being 1:2) in methanol for 5 h to deliver *N*-propargyl(pyrrolyl)aminoenones **54** (Scheme 30). The latter were formed as a mixture of *E/Z* isomers stabilized by intramolecular H-bonds between carbonyl group and NH-function of the amino moiety (the *Z-*isomer) or NH-function of the pyrrole ring (the *E*-isomer) with predominance of the *Z*-isomer.

The electronic nature of the substituents attached to the pyrrole ring determines the isomers ratio. Thus, for aminoenone **54** with unsubstituted pyrrole ring, the *Z/E* ratio is ~9:1. When a donor cyclohexane moiety is attached to the pyrrole ring [R^1^-R^2^ = (CH_2_)_4_], this ratio becomes 15:1, probably owing to a lower NH-acidity of the pyrrole counterpart and hence a weaker stabilization of the *E*-isomer by the intramolecular H-bonding. Consequently, for pyrroles with electron-withdrawing aryl substituents, having more acidic pyrrole NH-proton, the content of the *E-*isomer increases, *Z/E* ratio being ~4:1.

The cyclization of N-propargyl(pyrrolyl)aminoenones **54** was implemented by heating (60 °C, 15–30 min ) in the system Cs_2_CO_3_/DMSO to afford pyrazines **53a** with exocyclic double bond and their thermodynamically more stable endocyclic isomers **53b**. Pyrrolopyrazines **53b** with the endocyclic double bond were formed selectively only from aminoenones **54** with unsubstituted pyrrole ring or with tetrahydroindole derivatives. In the case of enaminones with phenyl or fluorophenyl substituents, the major products were pyrrolopyrazines having the exocyclic double bond **53a** (their content in the reaction mixture was spanned 70–90%), while pyrrolopyrazines **53b** were minor products. The total yield of both isomers remained almost quantitative (90–96%).

Later, pyrrolopyrazines **53a,b** were obtained (90–95% yields) via a one-pot procedure from ethynylpyrroles **52** and propargylamine when the reactants were heated (60–65 °C) in DMSO [77].

#### 5.1.2. Synthesis of Pyrrolyl Pyridines

**Scheme 31:**
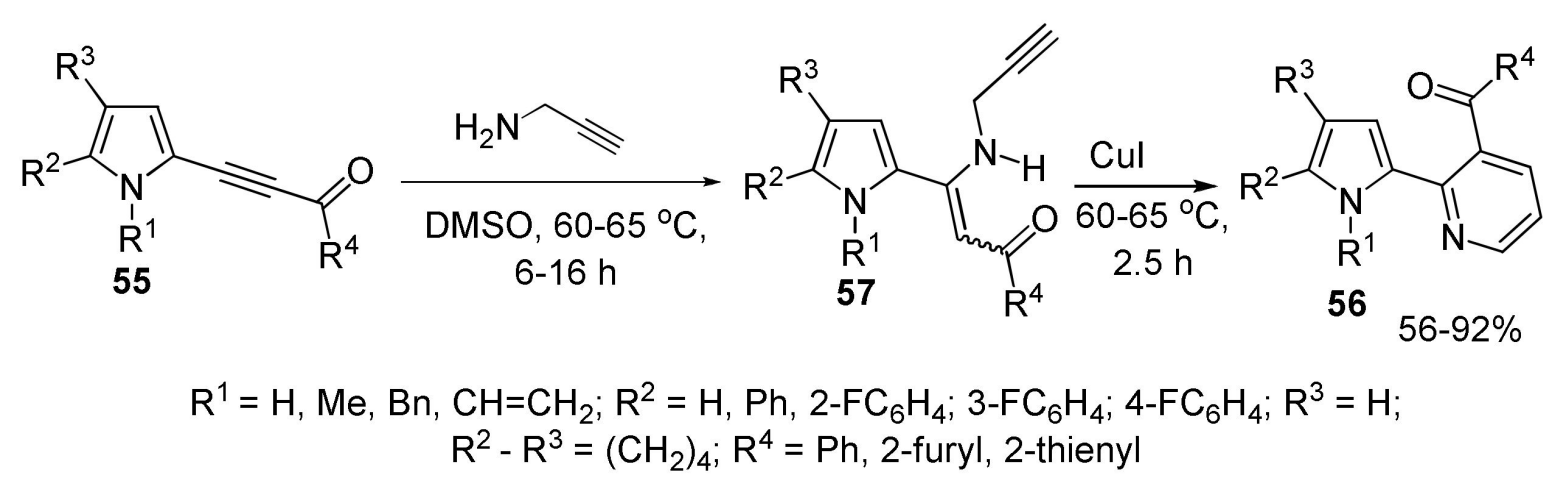
The formation of pyrrolyl pyridines **56** from acylethynylpyrroles and propargylamine.

The one-pot reaction of *N*-substituted acylethynylpyrroles **55** with propargylamine in the presence of CuI selectively afforded 2-(pyrrol-2-yl)-3-acylpyridines **56** (Scheme 31) [78].

Catalyst-free heating of the reactants led to *N*-propargyl(pyrrolyl)aminoenones **57** which, upon keeping with CuI (equimolar amount) for 2.5 h at the same temperature, underwent the dihydrogenative ring closure to give pyrrolyl pyridines **56** (Scheme 31).

The duration of non-catalytic step strongly depended on the pyrrole structure: the acceptor substituents in the pyrrole ring facilitated the reaction (the reaction time was 6 h), while the donor ones slowed down the process (the reaction time was 16 h). A peculiar feature of this dehydrogenative cyclization is that the intermediate dihydropyridines **58** were aromatized rapidly (they are not usually detectable in the reaction mixture). Only in the case of acylethynyltetrahydroindole dihydropyridine **58** was isolated in 4% yield. Notably, the catalytic ring closure was almost insensitive to the structure of the initial acylethynylpyrroles **55** (the reaction time was about 2.5 h for all the cases).

**Scheme 32:**
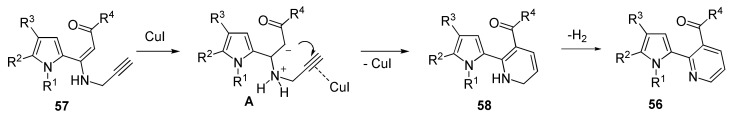
Formation of pyrrolyl pyridines **56** from aminoenones **57**.

A less predictable step of the synthesis is the intramolecular nucleophilic addition of the CH-bond adjacent to carbonyl group across the acetylenic moiety (Scheme 32). This CH-bond can be deprotonated under the action of amino group, either intramolecularly (autodeprotonation) to generate intermediate **A** or intermolecularly. Upon the complexing of Cu^+^ cation with the triple bond, the latter should be polarized to increase sensitivity towards the nucleophilic attack. This attack is completed by the addition of the carbanionic site to the terminal acetylenic atom to give the intermediate dihydropyridine **58**.

The MS spectra of the reaction mixtures showed that the oxidation of intermediate **58** did not take place under the action of DMSO (no Me_2_S was detected). The air oxygen also did not participate in this process: the same results were obtained both under argon blanket and on air. Therefore, the Cu^+^ cation was considered [78] as a likely oxidant.

**Scheme 33:**
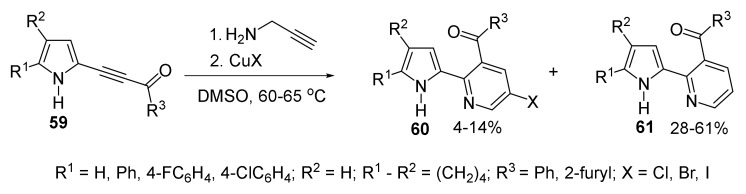
Synthesis of pyrrolyl pyridines **60** and **61** from NH-2-acylethynylpyrroles and propargylamine.

Latter [79] from the reaction of NH-acylethynylpyrroles **59** with propargylamine in the presence of CuX (X = Cl, Br, I), 3-acyl-2-(pyrrol-2-yl)-5-halopyridines **60** were unexpectedly isolated in 4–14% yields along with 3-acyl-2-(pyrrol-2-yl)pyridines **61** (28–61% yields) (Scheme 33). Evidently, the cause of this difference compared to the previous cyclization [78] was the NH-functionality of the starting acylethynylpyrroles.

**Scheme 34:**
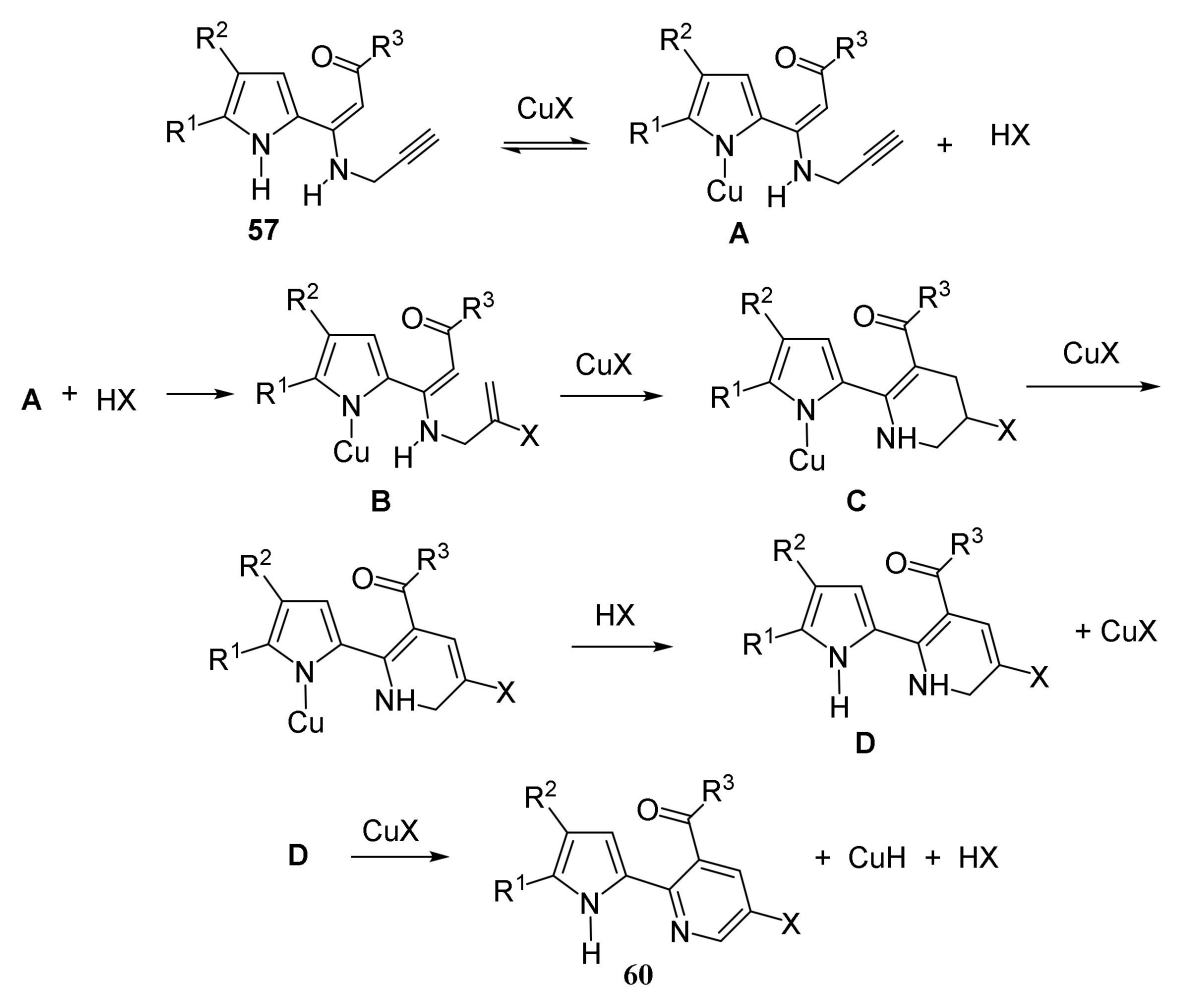
Proposed scheme of halopyridines **60** formation.

Under the above conditions, pyrrolyl pyridines **61** were not halogenated with CuX, thus indicating that construction of the halogenated pyridine ring occurred before its closure. It is supposed (Scheme 34) [79] that hydrogen halides, reversibly generated by the interaction of the NH pyrrole moiety of the intermediate *N*-propargyl(pyrrolyl)aminoenone **57** with CuX, add to the triple bond activated by π-complexing with other CuX molecules to give haloallyl intermediate **B** (Scheme 34). Afterwards, the intramolecular addition of the CH bond to the allyl moiety takes place to form the intermediate 5-halotetrahydropyridyl intermediate **C**. Aromatization of the latter is finalized via the reaction with CuX and further oxidation by Cu^+^ cations as previously described for a similar process [78].

### 5.2. Synthesis of Pyrrolizines via Three-Component Cyclization with Benzylamine and Acylacetylenes

**Scheme 35:**
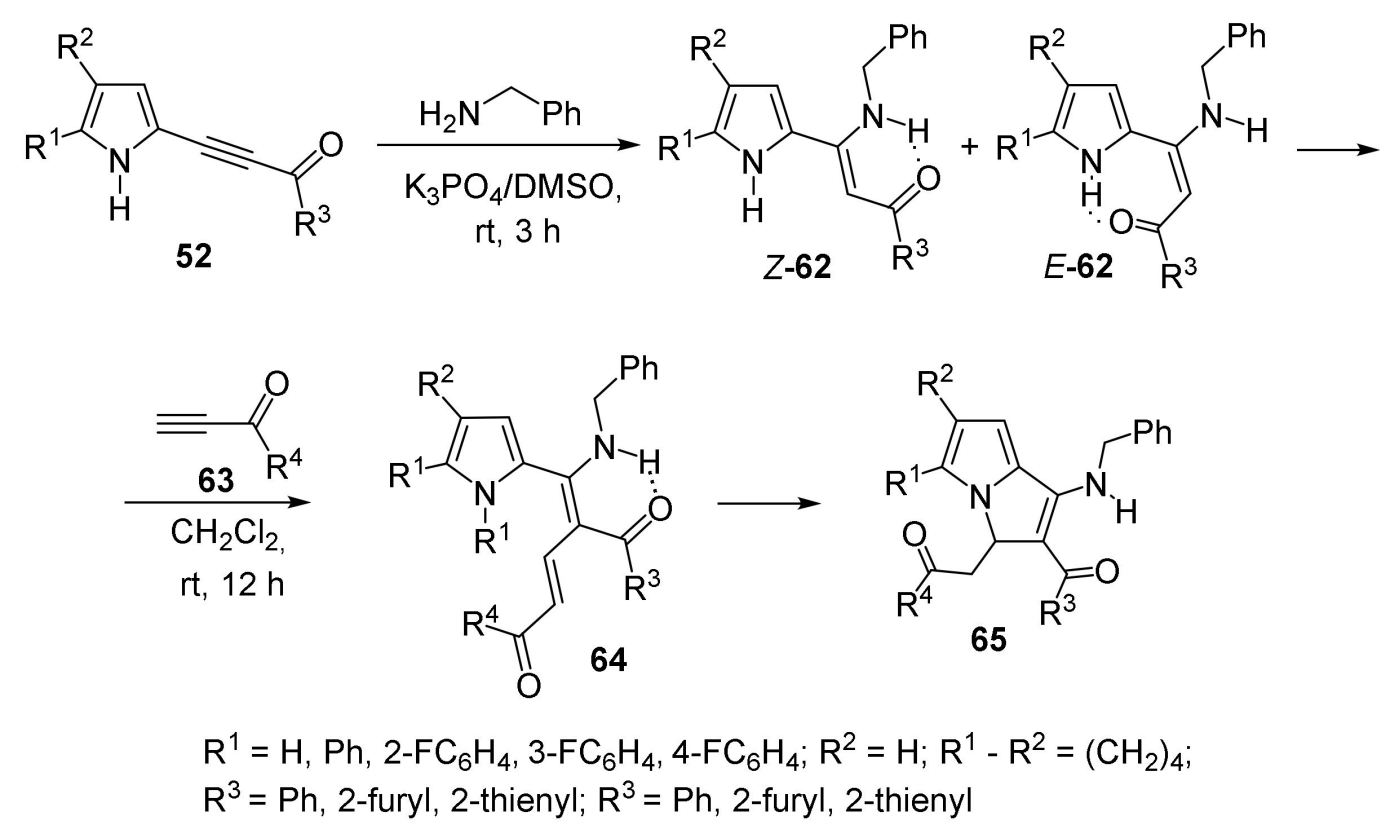
Synthesis of 1-benzylamino-2-acyl-3-methylenoacylpyrrolizines **65**.

On the platform of acylethynylpyrroles **52**, a new general strategy for the synthesis of functionalized pyrrolizines was developed [80]. It consisted of the two steps: (i) the base-catalyzed addition of a benzylamine to 2-acylethynylpyrroles **52** to give pyrrolylaminoenones **62**; (ii) non-catalyzed addition of *N*-benzyl(pyrrolyl)aminoenones **62** to the triple bond of acylacetylenes **63** followed by the intramolecular cyclization of the intermediate pentadiendiones **64** thus formed to 1-benzylamino-2-acyl-3-methylenoacylpyrrolizines **65** (Scheme 35).

The nucleophilic addition of benzylamine to the triple bond of 2-acylethynylpyrroles **52** was realized in the presence K_3_PO_4_/DMSO catalytic system to smoothly deliver *N*-benzyl(pyrrolyl)aminoenones **62** in up to 97% yield (Scheme 35). The latter were formed as a mixture of the *E/Z* isomers, the *E*-isomer being obviously stabilized by intramolecular H-bonds between the carbonyl group and NH-function of the pyrrole ring. As in the case of the addition of propargylamine to acylethynylpyrrole (see Section 5.1), the structure of the substituents of the pyrrole ring strongly influences the isomer ratio of the adducts: the donor substituents increase the content of the Z-isomers.

Further, the aminoenones **62** chemo- and regioselectively reacted with acylacetylenes **63** to afford the intermediate pentadiendiones **64**, which then cyclized to 1-benzylamino-2-acyl-3-methylenoacylpyrrolyzines **65** in up to 80% yield (Scheme 35).

### 5.3. Reactions with Ethylenediamine

**Scheme 36:**
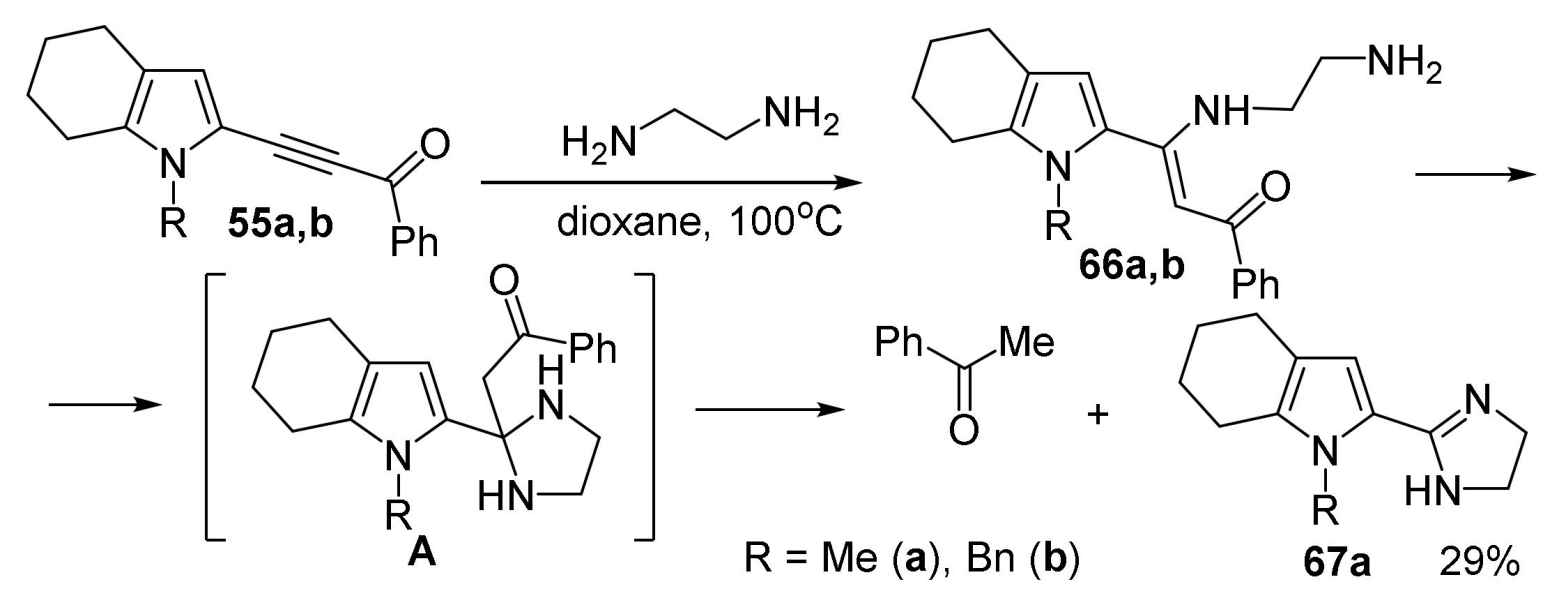
Reaction of 2-benzoylethynylpyrroles **55a,b** with ethylenediamine.

The reaction of 2-benzoylethynylpyrroles **55a**,**b** with ethylenediamine was realized upon reflux of their equimolar mixture in dioxane (40 h) [81]. Expectedly, first the addition of diamine gave monoadduct **66a**,**b**, which, in the case of acylethynylpyrrole **55a**, underwent intramolecular cyclization/fragmentation to afford tetrahydroindolyl imidazoline **67a** and acetophenone (Scheme 36).

**Scheme 37:**
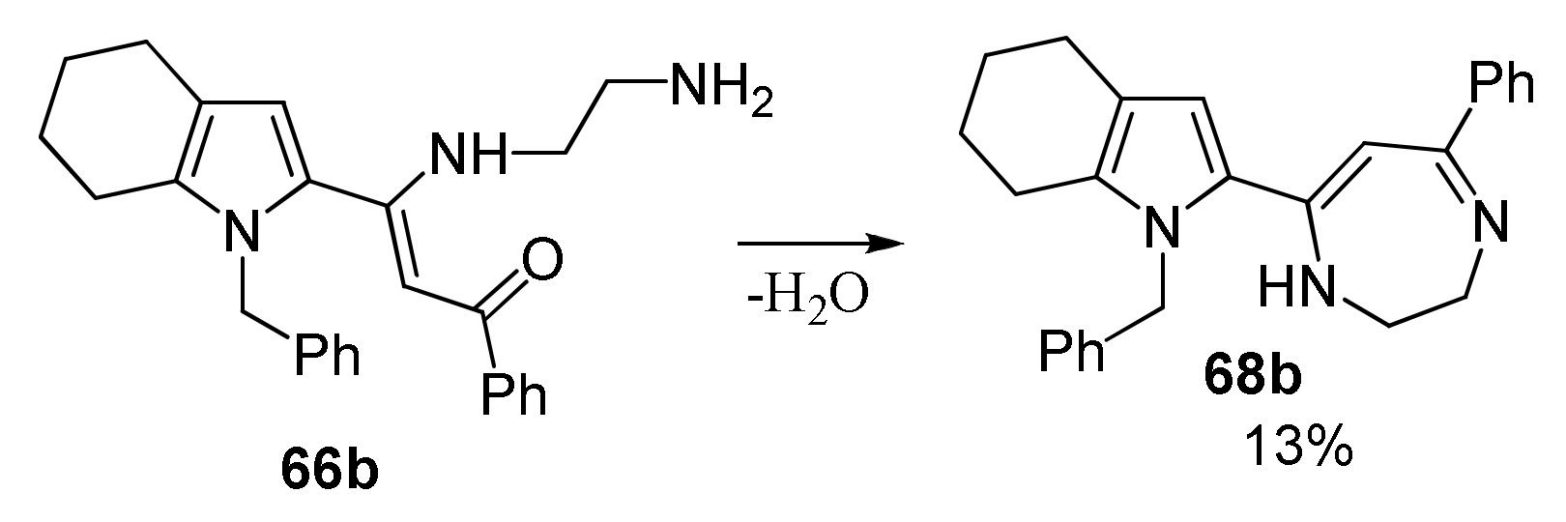
The formation of tetrahydroindolyl dihydrodiazepine **68b.**

In this reaction, in the case of acylethynylpyrrole **55b**, the formation of dihydrodiazepine **68** takes place. This is a result of the intramolecular cyclization of monoadduct **66b** with the participation of the carbonyl group followed by dehydration (Scheme 37).

### 5.4. Cyclization with Hydrazine: Synthesis of Pyrrolyl Pyrazoles

**Scheme 38:**
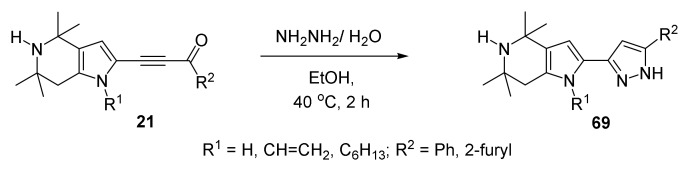
Synthesis of 4,5,6,7-tetrahydropyrrolo[3,2-c]pyridine-pyrazole ensembles **69**.

The building up of the pyrazole ring over acetylenic moiety of pyrrolopyridine propynones **21** via its ring closure with hydrazine gave 4,5,6,7-tetrahydropyrrolo[3,2-*c*]pyridine-pyrazole ensembles **69** in 92–98% yields (Scheme 38) [59].

**Scheme 39:**
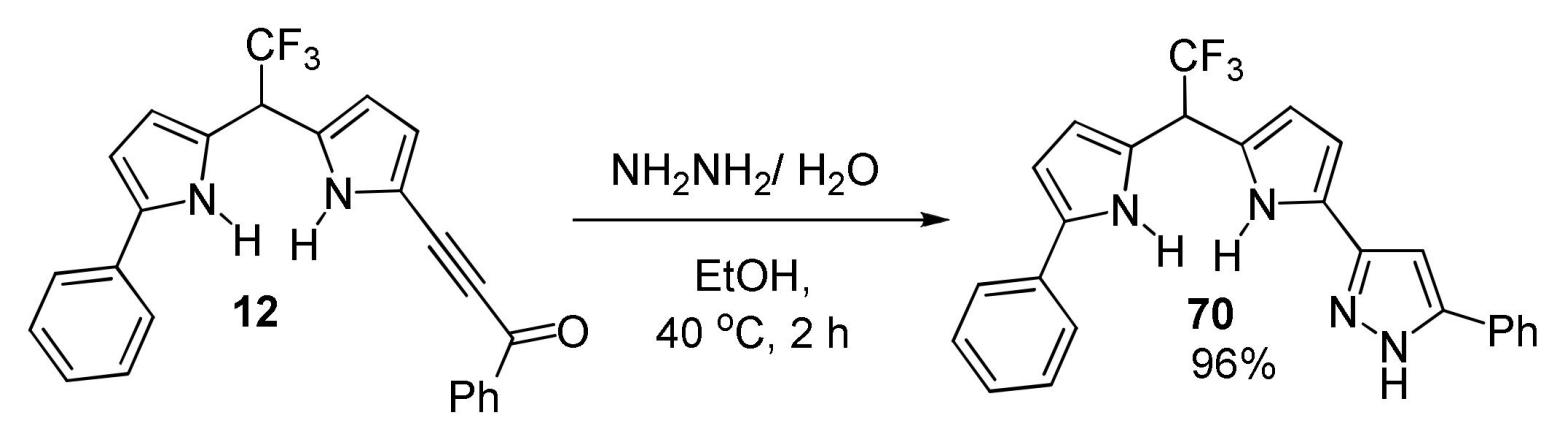
Synthesis of dipyrromethane-pyrazole ensemble **70**.

According to the above procedure, a new extended dipyrromethane system conjugated with pyrazole cycle **70** was obtained in almost quantitative yield (Scheme 39) [57].

### 5.5. Cyclization with Hydroxylamine: Synthesis of Pyrrolyl Isoxazoles

**Scheme 40:**
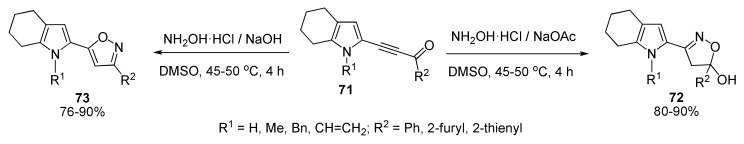
Reaction of acylethynyltetrahydroindoles **71** with hydroxylamine.

Acylethynyltetrahydroindoles **71** readily cyclized with hydroxylamine to give regioselectively either 3-(4,5,6,7-tetrahydroindol-2-yl)-4,5-dihydroisoxazol-5-ols **72** or 5-(4,5,6,7-tetrahydroindol-2-yl)isoxazoles **73** (Scheme 40) [82]. The cyclization can be easily switched from the direction leading exclusively to isoxazoles **72** to the formation of isoxazoles **73** by simple changing of the proton concentration in the reaction mixture. When the reaction was carried out in the presence of acetic acid (NH_2_OH·HCl/NaOAc, 1:1 system), only isoxazoles **72** were formed, whereas under neutral or basic conditions (NH_2_OH·HCl/NaOH (1:1 or 1:1.5 system), the cyclization took another pathway to produce preferably (94s–97% or entirely) isoxazoles **73**.

**Scheme 41:**
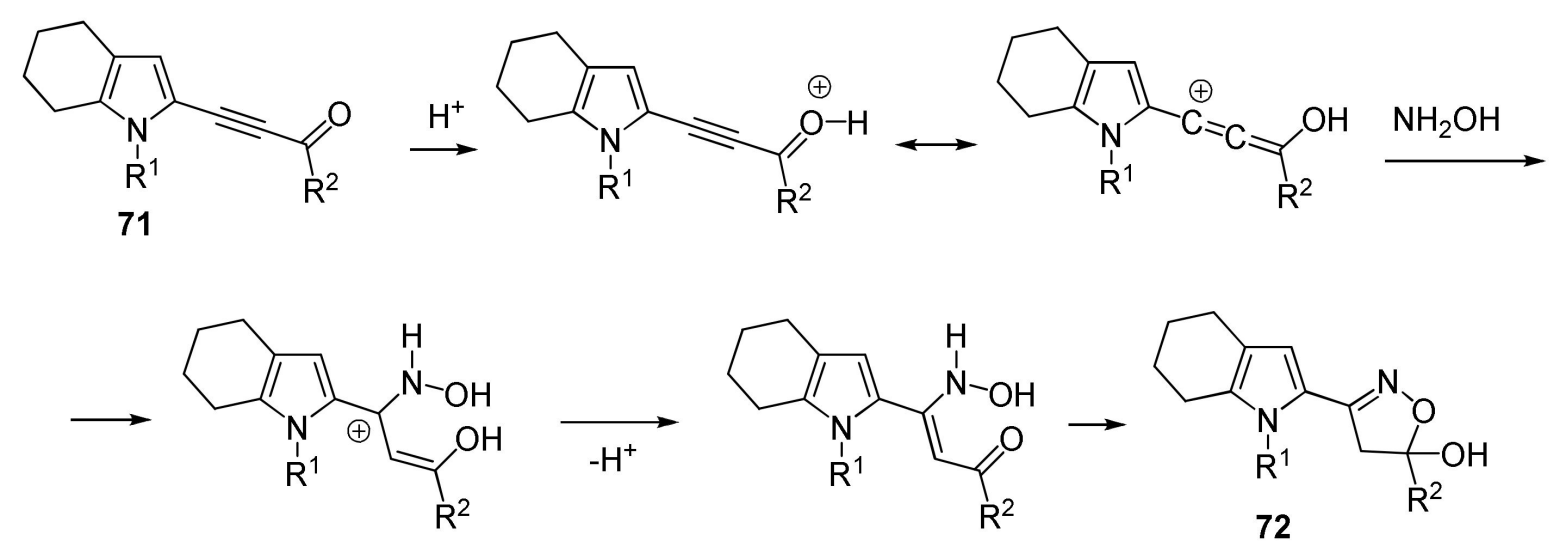
The formation of 3-(4,5,6,7-tetrahydroindol-2-yl)-4,5-dihydroisoxazol-5-ols **72**.

Apparently, in the presence of acetic acid, the attack of the NH_2_OH nucleophile at the β-acetylenic carbon of tetrahydroindoles **71** is electrophilically assisted by the simultaneous protonation of the carbonyl group (and finally 1,4-addition takes place to deliver isoxazoles **72**), as shown in Scheme 41.

In the presence of the NH_2_OH·HCl/NaOH system, which is unable to exert the electrophilic assistance, the common oximation of the carbonyl group prevailed.

**Scheme 42:**
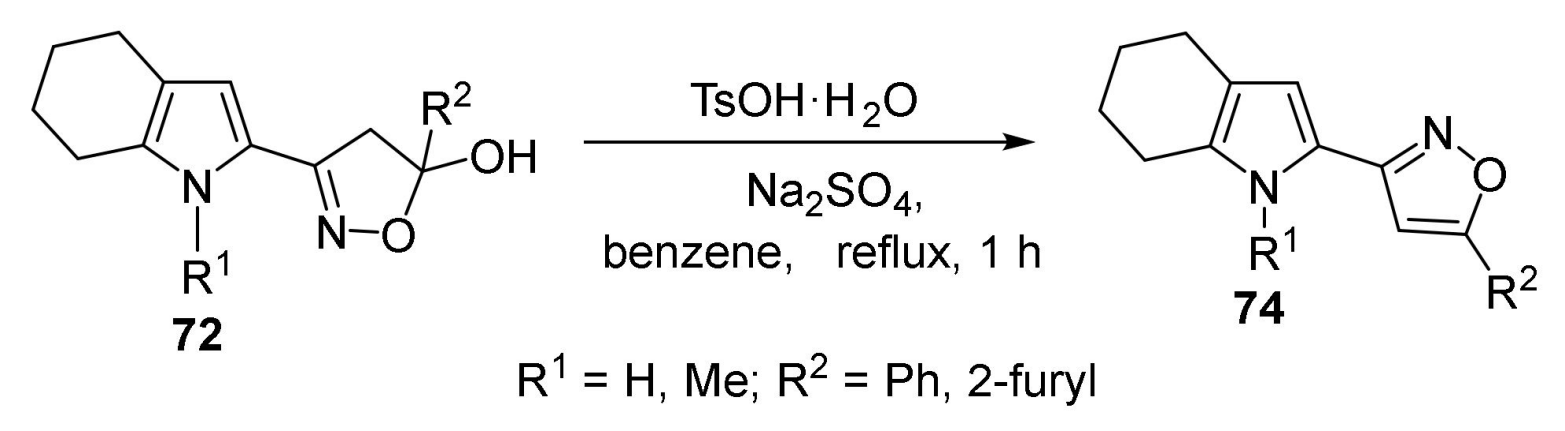
Dehydration of 3-(4,5,6,7-tetrahydroindol-2-yl)-4,5-dihydroisoxazol-5-ols **72**.

Moreover, 4,5-dihydroisoxazol-5-ols **72** underwent easy aromatization when refluxing (benzene, 1 h) in the presence of TsOH·H_2_O to isoxazoles **74** in 73–91% yields (Scheme 42) [82].

On the basis of the above cycloaddition, two approaches to the synthesis of *meso*-CF_3_ substituted dipyrromethanes **75–77** bearing isoxazole moieties were developed [83].

**Scheme 43:**
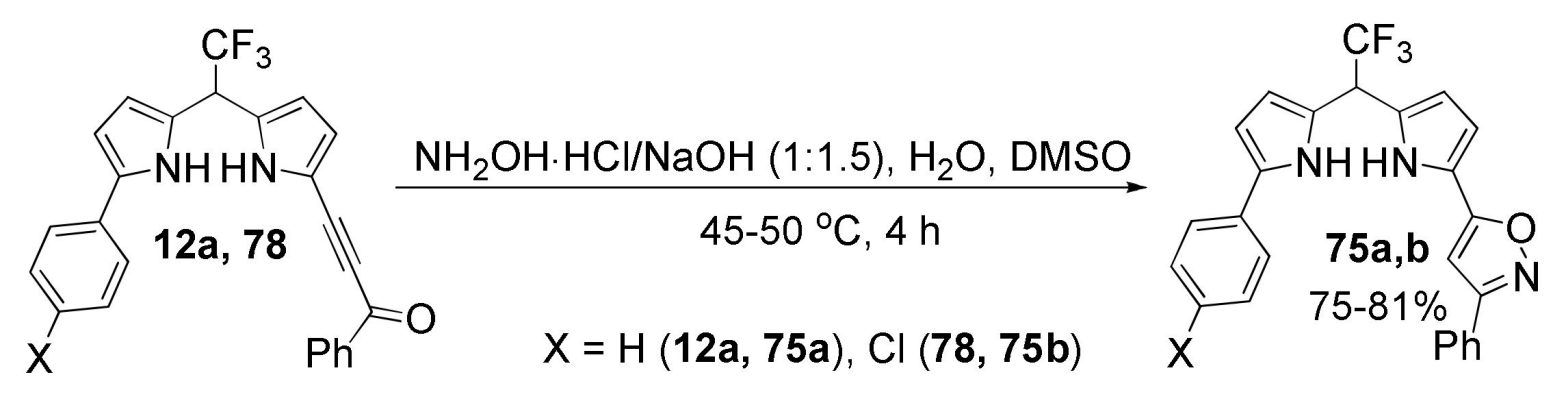
Synthesis of (3-phenylisoxazol-5-yl)dipyrromethanes **75a,b** from ethynyldipyrromethanes **12**, **78**.

**Scheme 44:**
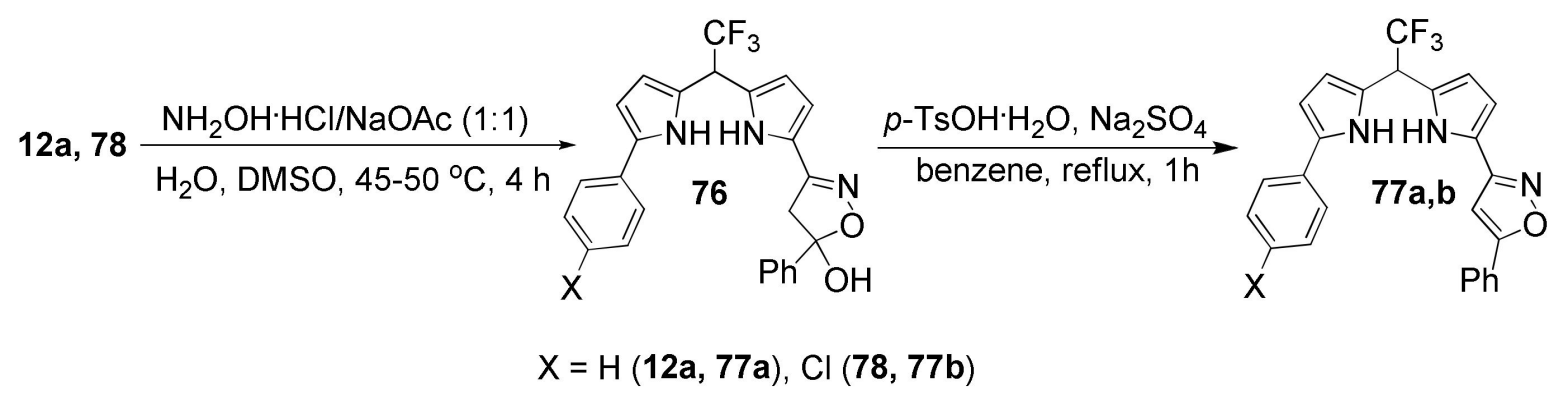
Synthesis of (5-phenylisoxazol-3-yl)dipyrromethanes **77a,b** from ethynyldipyrromethanes **12**, **78**.

**Scheme 45:**
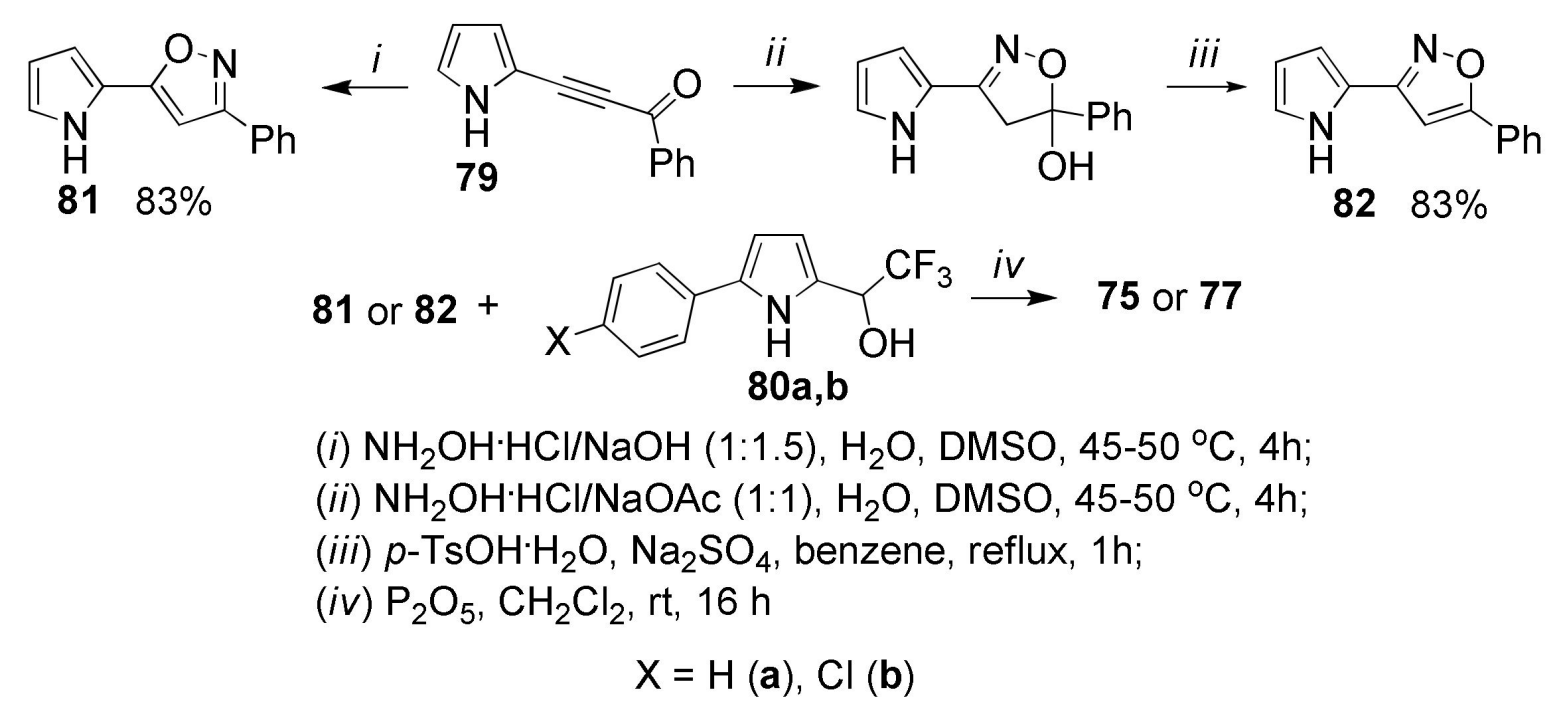
Alternative synthesis of (3- or 5-phenylisoxazolyl)dipyrromethanes **75** and **77** by condensation of pyrrolylisoxazoles **81** or **82** and with 2,2,2-trifluoro-1-(pyrrol-2-yl)-1-ethanols **80**.

The key stages of these approaches are the cycloaddition of hydroxylamine to the triple bond of ethynyldipyrromethanes **12a**, **78** (Scheme 43 and Scheme 44), or the synthesis of pyrrolyl isoxazoles **75**, **77** from ethynylpyrrole **79** (accessible from pyrrole and benzoylbromoacetylene), and its further condensation with 2,2,2-trifluoro-1-(pyrrol-2-yl)-1-ethanols **80**, as described in Section 2.1.2. (Scheme 45).

### 5.6. Cyclization with Methylene Active Esters: Synthesis of Pyrrolyl Pyrones

**Scheme 46:**
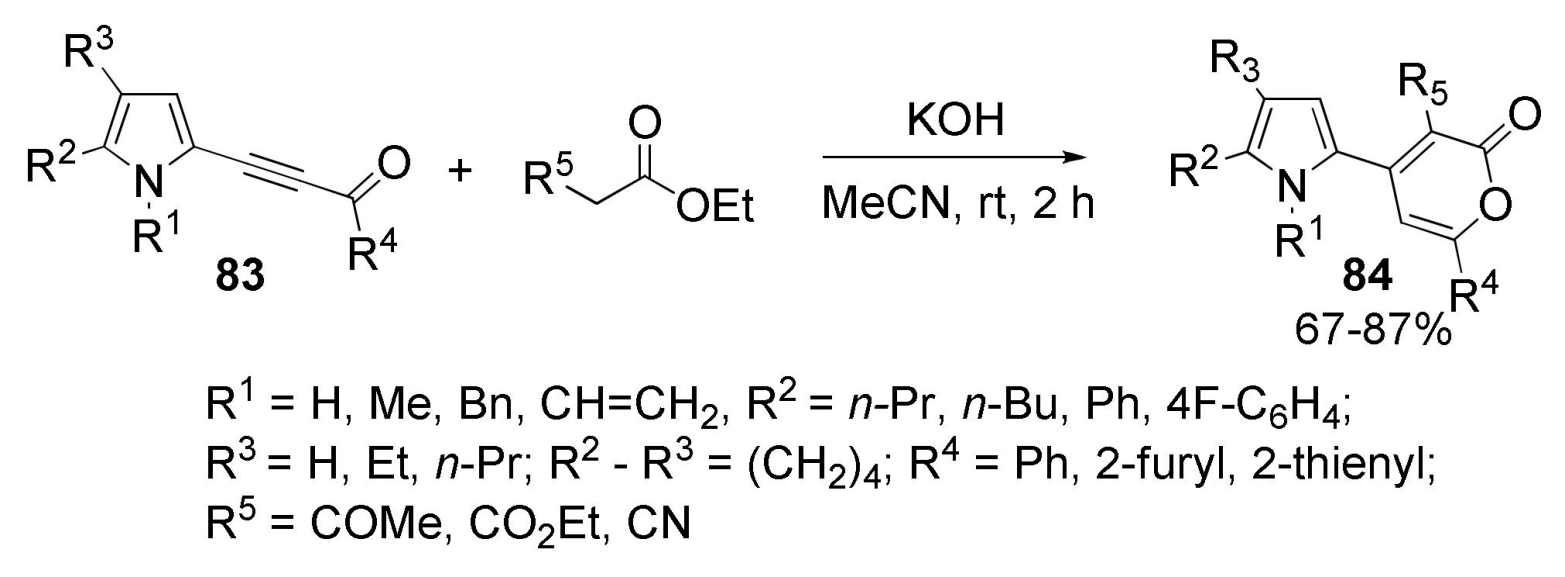
The synthesis of pyrrolyl pyrones **84**.

The [4+2]-cycloaddition between 2-acylethynylpyrroles **83** and methylene active esters (Scheme 46), offering a short-cut to pyrrolyl pyrones **84** in good to high yields, was described [84].

The reaction was carried out in acetonitrile in the presence of 1.5 molar excess of KOH. As methylene active esters, diethylmalonate, ethyl acetoacetate and ethyl cyanoacetate were used.

**Scheme 47:**
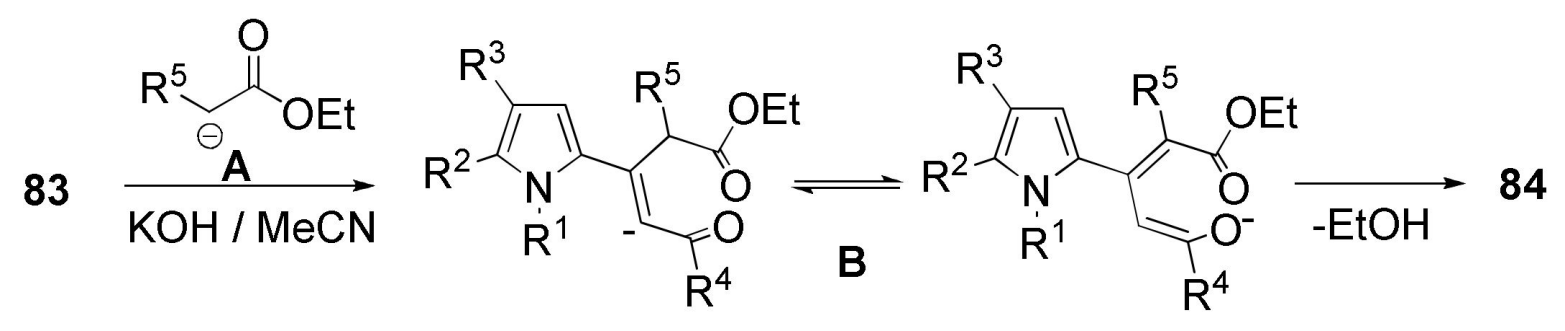
Scheme of pyrrolyl pyrones **84** formation.

The cyclization is triggered by the proton abstraction from the active CH_2_ group of methylene active esters followed by the nucleophilic attack of the carbanion **A**, thus generated at the triple bond of acylethynylpyrroles **83** to afford intermediate **B**. The subsequent intramolecular nucleophilic substitution of the ethoxy group in the ester function by the oxygen-centered anion (the resonance form of the intermediate **B**) furnishes the target products (Scheme 47).

### 5.7. Unprecedented Four-Proton Migration in Acylethynylmenthofurans: “A Proton Pump”

**Scheme 48:**
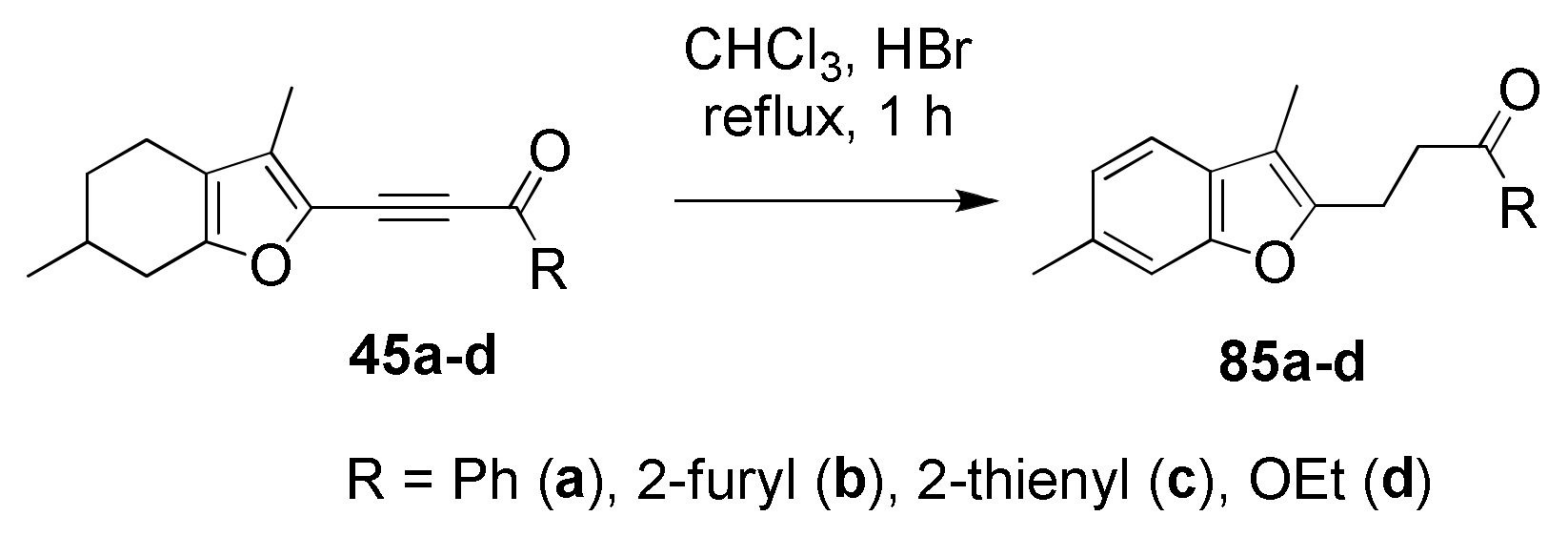
Rearrangement of acylethynylmenthofurans **45** to acylethylbenzofurans **85**.

When benzoylethynylmenthofuran **45a** was heated at reflux in CHCl_3_ in the presence of HBr, the formation of benzoylethylbenzofuran **85a** in 95% yield was observed (Scheme 48) [85]. Thus, the transfer of four hydrogen atoms from the cyclohexane ring to the triple bond took place.

This rearrangement was found to be general for other acylethynyl derivatives (furoyl, thenoyl, alkoxycarbonyl) of menthofuran to give their acylethylbenzofuran derivatives in the yield of 44%, 48%, and 24% respectively (Scheme 48).

**Scheme 49:**
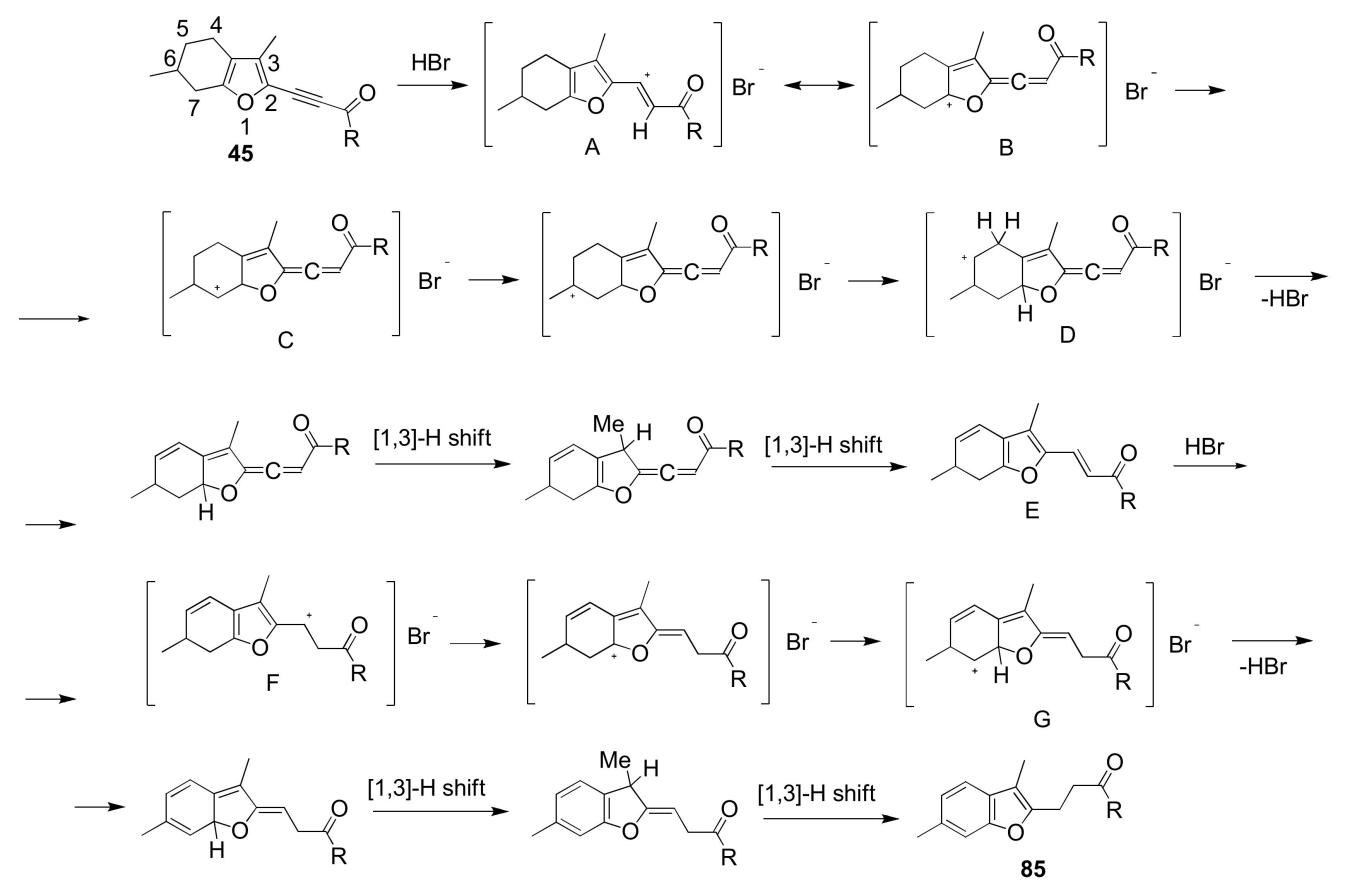
Proposed mechanism for the transfer of four hydrogens.

Basing on these experimental results, it can be postulated that the rearrangement starts with protonation of acylethynyltetahydrobenzofuran moiety with HBr to give carbocation **A**, which in its more stable mesomeric form **B** abstracts a hydride-ion from the adjacent position (C-7) with positive charge transfer to form carbocation **C**. Then, two hydride shifts in the cyclohexane ring transform carbocation **C** into carbocation **D** with the positive charge at C-5. Proton abstraction from the C-4 position of this carbocation leads to the cyclohexene moiety and regenerates HBr. Simultaneously, after two 1,3-hydrogen shifts in the furan counterpart, it is transformed into vinyl intermediate **E**. Next, protonation of the double bond with HBr results in the formation of carbocation **F** which in its stable endocyclic form accepts the hydride ion from the cyclohexene ring to give cyclohexene carbocation **G**. The release of a proton from the latter gives the cyclohexadiene ring and HBr. Two 1,3-hydrogen shifts in the furan moiety completes the four-hydrogen transfer to the side chain giving 3,6-dimethylbenzofuran **87** with a saturated side chain, i.e., an exhaustively hydrogenated acetylene moiety (Scheme 49).

The driving force of this spectacular “hydrogen pump” is the energy gain due to the formation of the aromatic benzofuran system.

## 6. Concluding Remarks and Outlook

This review evidences that the cross-coupling reactions between electrophilic haloacetylenes and electron-rich heterocycles assisted by Al_2_O_3_ or K_2_CO_3_ or similar solid oxides and salts continue to be expanded, occupying more and more areas of heterocyclic chemistry. These endeavors are stimulated by such competitive beneficial features of this methodology as transition metal-free, no-solvent, mild conditions, availability of the starting materials, very simple synthetic operations, and possibility to introduce acetylenic substituents with electron-withdrawing groups into a heterocyclic core. Now, these reactions pave a short way to previously inaccessible or unknown, highly reactive heterocyclic building blocks and precursors to create novel heterocyclic systems of greater diversity and complexity.
